# Abyssomicin Biosynthesis: Formation of an Unusual Polyketide, Antibiotic-Feeding Studies and Genetic Analysis

**DOI:** 10.1002/cbic.201100172

**Published:** 2011-06-09

**Authors:** Elvira M Gottardi, Joanna M Krawczyk, Hanna von Suchodoletz, Simone Schadt, Agnes Mühlenweg, Gabriel C Uguru, Stefan Pelzer, Hans-Peter Fiedler, Mervyn J Bibb, James E M Stach, Roderich D Süssmuth

**Affiliations:** [a] Technische Universität Berlin, Institut für ChemieStrasse des 17. Juni 124, 10623 Berlin (Germany), Fax: (+49) 30-314-79651; [b] School of Biology, Newcastle UniversityNewcastle-upon-Tyne, NE1 7RU (UK); [c] B.R.A.I.N. AktiengesellschaftDarmstädter Strasse 34, 64673 Zwingenberg (Germany); [d] Dept. of Microbiology/Biotechnology, Universität TübingenAuf der Morgenstelle 28, 72076 Tübingen (Germany); [e] Department of Molecular Microbiology, John Innes CentreNorwich, NR4 7UH (UK)

**Keywords:** antibiotics, biosynthesis, feeding studies, gene clusters, polyketides

Abyssomicin C, produced by the marine actinomycete *Verrucosispora maris* AB-18-032, is active against Gram-positive bacteria including methicillin-resistant *Staphylococcus aureus* (MRSA) and inhibits *p*-aminobenzoate formation during tetrahydrofolate synthesis; it is the first natural product active against this therapeutic target. To investigate the biosynthesis of this small but structurally complex secondary metabolite, we carried out feeding studies using ^13^C labelled polyketide building blocks. Formation of abyssomicin C requires two propionates, five acetates and one glucose-derived metabolite. Identification and sequencing of the abyssomicin biosynthetic gene cluster revealed a 57 kb segment of *Verrucosispora maris* AB-18-032 DNA that contained all of the genes necessary for abyssomicin biosynthesis. The identity of the biosynthetic gene cluster was confirmed by gene inactivation and complementation experiments (the first genetic manipulation of a member of this genus) and a model for abyssomicin C biosynthesis is proposed.

The search for novel antibiotics has led to the exploration of previously unscrutinised and extreme habitats, such as the deep oceans, caves, deserts and mountains, to isolate new microbial strains with untapped biosynthetic potential.^1^, ^2^ Concomitantly, rational approaches have identified new therapeutically useful bacterial targets.^2^, ^3^ The folate pathway is essential in bacteria for the synthesis of aromatic amino acids and purine nucleotides, and plays an essential role in pathogenesis. The pathway is also present in algae, higher plants, fungi and apicomplexan parasites, but absent from mammals, and is, thus, an excellent potential target for the development of new therapeutic compounds.^4^ So far, only synthetic drugs are known to inhibit proteins in this pathway; foremost are the sulfonamides (sulfa drugs) and diaminobenzylpyrimidines (e.g., trimethoprim).^5^ In 2004, targeted screening for inhibitors of the folate pathway led to the discovery of abyssomicins produced by the marine actinomycete *Verrucosispora maris* AB-18-032.^6^ Abyssomicin C is active against Gram-positive bacteria, including MRSA,^6^ and inhibits the biosynthesis of *p*-aminobenzoic acid (pABA), a constituent of the folate pathway.^7^ Recently, we found that abyssomicin C covalently binds to PabB, the aminodesoxychorismate synthase of *Bacillus subtilis*, and inhibits pABA formation and, consequently, folate biosynthesis.^7^, ^8^

Several strategies for the synthesis of abyssomicin C have been published and provide valuable new insights into the biosynthesis and activity of abyssomicin C.^9^ The biomimetic synthesis by Sorensen et al.9d provides an interesting model for the biosynthesis of abyssomicin C. In 2007, while working on another route for total synthesis of abyssomicin C, the Nicolaou group discovered *atrop*-abyssomicin C, the *atrop*-isomer of abyssomicin C. *Atrop*-abyssomicin C is even more active than the initially reported abyssomicin C.^7^, 9a, 10 Shortly thereafter, *atrop*-abyssomicin C (**2**) was also detected in fermentations of *Verrucosispora maris* AB-18-032.^7^ In fact, **2** is the main product synthesised by *Verrucosispora maris* AB-18-032, and abyssomicin C (**1**) is a minor by-product formed from **2** by rearrangement under acid conditions. To date, seven abyssomicins have been identified but only abyssomicin C and *atrop*-abyssomicin C have defined bioactivity.^7^, ^8^

*Atrop*-abyssomicin C and abyssomicin C are structurally related to tetronomycin-like antibiotics, such as chlorothricin,^11^ tetronomycin and tetrocarcin, which all contain a tetronic acid moiety (4-hydroxy-(5*H*)furan-2-one). The abyssomicins contain an oxo-bridge from the tetronate that forms a bicyclic system, which is unique among the tetronomycin-like antibiotics. Tetronic acid antibiotics are an important subclass of polyketide antibiotics with various bioactivities and molecular targets, and the biosynthetic gene clusters of several have been elucidated: chlorothricin, kijanimicin, tetronomycin and tetrocarcin.^12^ Comparison of these gene clusters was used to identify a unique set of genes required for the biosynthesis of tetronic acid-containing compounds.12b In this work, we propose a model for the biosynthesis of *atrop*-abyssomicin C based on a combination of feeding studies with ^13^C-labelled biosynthetic precursors and the identification of the abyssomicin biosynthetic gene cluster. We propose that abyssomicin is synthesised as a linear polyketide chain from five acetates, two propionates and a metabolite from the glycolytic pathway. Polyketide synthase (PKS) processing is followed by formation of the tetronate moiety, a Diels–Alder reaction, and oxygenation.

By analogy to other tetrocarcin-type antibiotics, it was assumed that the abyssomicins are formed by a polyketide biosynthetic pathway.3a To confirm this assumption, the ^13^C-labelled building blocks [1-^13^C]acetate, [1,2-^13^C]acetate and [1-^13^C]propionate were fed to exponentially growing shake-flask cultures of *Verrucosispora maris* AB-18-032. Once the cultures had reached stationary phase, *atrop*-abyssomicin C was isolated, as described previously, and analysed by ^13^C NMR spectroscopy.3a The incorporation of ^13^C carbons into the polyketide backbone resulted in a considerable increase in signal intensity of the labelled carbon positions. Feeding of [1-^13^C]acetate led to signal increase at C-1, C-7, C-9, C-11 and C-13 (Scheme 1). In a subsequent experiment, [1,2-^13^C]acetate was fed, and intact incorporation was found for C-1/C-2, C-7/C-8, C-9/C-10 and C-11/C-12. The resulting doublets with their ^13^C–^13^C coupling constants as well as all other ^13^C NMR data are given in Table S1 in the Supporting Information.

**Scheme 1:**
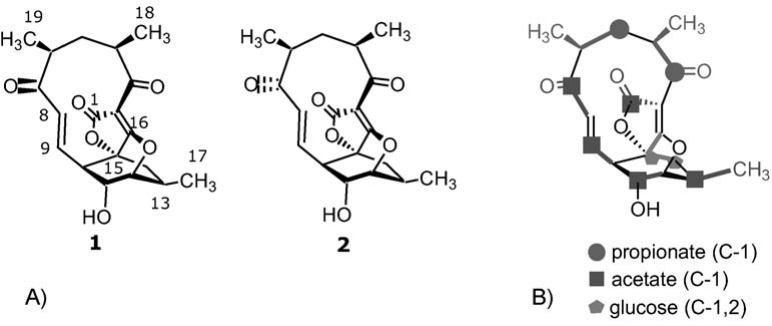
A) Structures of the antibacterial polyketides abyssomicin C (**1**) and *atrop*-abyssomicin C (**2**). B) Incorporation of ^13^C-labelled precursors into the polyketide backbone of abyssomicin C.

This pairwise ^13^C–^13^C coupling is indicative of direct assembly into the polyketide chain. When [1-^13^C]propionate was fed, increased signals were obtained for C-3 and C-5. The assembly of five acetate and two propionate units as biosynthetic precursors into the abyssomicin C carbon skeleton leaves a three carbon unit (C-14–C-16) unassigned. Since earlier studies on tetronic acid antibiotics had reported the incorporation of glycerol at this position,^13^ [1-^13^C]glycerol was added to the fermentation broth of *Verrucosispora maris* AB-18-032. Subsequently isolated *atrop*-abyssomicin C was analysed by ^13^C NMR spectroscopy, but no incorporation of glycerol was detected. Likewise, feeding of other putative ^13^C-labelled C_3_ precursors, such as serine, alanine and succinic acid, did not yield specifically labelled *atrop*-abyssomicin C.^14^ Remarkably, only [1,2-^13^C]glucose was successfully incorporated into the C_3_ unit of the abyssomicin carbon backbone; this indicates that it originated from the glycolytic pathway. The ^13^C–^13^C coupling of C-14 and C-15 (*J*=36.5 Hz) clearly shows that C-14 and C-15 are derived from the same glucose molecule. Furthermore, while glucose leads to doubly labelled acetate (for C-3 through C-6 and C-18 and C-19) propionate is formed from only one ^13^C carbon, indicated by singlet peaks (Figures S1–S6 and Table S1 in the Supporting Information). This implies that in *Verrucosispora*, glycolysis preferentially ends in acetate14b rather than lactate14b and leads directly to propionate. In summary, feeding experiments showed that abyssomicin C is derived from five acetates, two propionates and one metabolite from the glycolytic pathway (Scheme 1).

A dual cosmid and whole genome sequencing (WGS) approach was used to identify the abyssomicin biosynthetic gene cluster (*aby*). A cosmid library of *Verrucosispora maris* AB-18-032 genomic DNA was hybridised with PKS type I-specific probes generated by PCR with primers KSIIFOR and ATIREV developed at Combinature Biopharm, AG.^15^ To identify which of the 49 hybridising cosmids contained genes responsible for *atrop*-abyssomicin C production, all were digested with two different restriction enzymes (BamHI, SmaI) and the resulting restriction profiles were used to classify the cosmids into families containing overlapping sequences. Three major families (with five to eight members each) and four smaller families (two members each) were identified, as well as 14 apparently unique cosmids. Representative cosmids from each family were subjected to restriction digestion with BamHI and the resulting fragments were cloned into the nonreplicating vector pK18mob2. After being sequenced, PKS positive clones were used for gene inactivation in *Verrucosispora* by selecting for single crossover integration into the host genome.

The resulting *Verrucosispora maris* AB-18-032 insertional mutants were screened for the absence of *atrop*-abyssomicin C production by analytical HPLC-ESI-MS, which revealed that cosmid family II contained genes required for abyssomicin C biosynthesis. One cosmid from this family (C17) was sequenced. Cosmids containing genomic segments overlapping that of C17 were identified by Southern analysis of restriction digests of members of cosmid family II by using *abyA2* (upstream of *abyB*; i.e., PKS genes) and *abyF1* (downstream of *abyB* genes) specific probes. Cosmids C49 and C45 were identified by using probes *abyA2* and *abyF1*, respectively, and sequenced to expand the extent of the *aby* gene cluster. Blastx searches of the WGS contig library were then carried out by using sequences from *abyX* (upstream) and *abyT* (downstream) to identify flanking regions. WGS contigs containing these genes and additional sequences were included in the assembly with data from cosmids C17, C45 and C49. This resulted in about 57 kb of contiguous genomic DNA sequence that appeared to contain the entire *aby* biosynthetic gene cluster (Figure 1).

**Figure 1:**
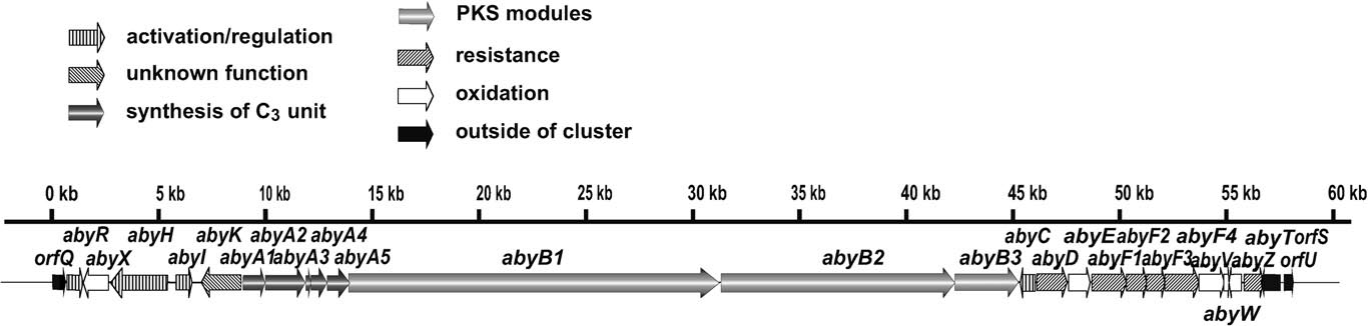
Biosynthetic gene cluster of *atrop*-abyssomicin C and assignment of the main gene functions.

Bioinformatics analysis of the 57 kb sequence with Glimmer and blastx revealed 24 putative protein coding sequences (pcs) that appeared to be involved in abyssomicin biosynthesis (Table 1 and Figure 1). These pcs comprise the anticipated PKS I genes (*abyB1*, *abyB2* and *abyB3*) and five genes (*abyA1*–*A5*) homologous to *chlM* and *chlD1*–*4* of the chlorothricin biosynthetic gene cluster involved in the assembly of the tetronic acid moiety.12c Genes encoding oxygenases, as well as genes with putative regulatory and export functions, were also identified.

**Table 1:** The abyssomicin biosynthetic gene cluster with functions assigned by xblast and InterProScan searches.

Name	Gene start [bp]	Length [aa]	Proposed function	Similarity with	Identity/ similarity [%]
*orfQ*	1	208	NUDIX hydrolase	*Frankia* sp. EAN1pec	53/66
			putative MutT family protein	*Nocardia farcinica* IFM 10152	43/52
*abyR*	680	248	transcriptional regulator, SARP family	[*Frankia* sp. *EUN1f*]	54/71
				*Frankia s*ymbiont of *Datisca glomerata*	58/69
*abyX*	2629	396	cytochrome P450	*Frankia* sp. EAN1pec *Streptomyces griseolus*	66/77 56/70
*abyH*	5389	889	LuxR family transcriptional regulator	*Frankia alni* ACN14a	33/46
			putative activator	*S. carzinostaticus*	35/45
*abyI*	5745	252	transcriptional regulator, SARP family	*S. longisporoflavus*	50/64
			activator protein	*S. hygroscopicus* ATCC 53653	48/63
*abyK*	8827	579	YD repeat	*Frankia* sp. EAN1pec	46/57
			RHS repeat-containing protein	*Amycolatopsis mediterranei* U32	36/50
*abyA1*	8911	341	β-ketoacyl-acyl-carrier protein synthase I	*Frankia* sp. EAN1pec	68/81
			3-oxoacyl-(acyl-carrier protein) synthase III	*Streptomyces* sp. NRRL 11266	64/75
*abyA2*	9933	622	phosphatase and glyceryl transferase	*Streptomyces* sp. NRRL 11266	57/68
			ChlD1	*S. antibioticus*	57/68
*abyA3*	11 798	78	discrete ACP	*Streptomyces* sp. NRRL 11266	58/75
			ChlD2	*S. antibioticus*	53/72
*abyA4*	12 031	251	dehydrogenase catalytic domain-containing protein	*Frankia* sp. EAN1pec	64/78
			pyruvate/2-oxogluatarate dehydrogenase	*Streptomyces* sp. NRRL 11266	60/73
*abyA5*	12 783	355	hydrolase superfamily dihydrolipoamide acyltransferase-like protein	*Actinomadura kijanata*	54/68
			ChlD4	*S. antibtioticus*	51/65
*abyB1*	13 847	5781	PKS I (module 1: KS, ATa, ACP; module 2: KS^Q^, ATa, DH, KR, ACP; module 3: KS, ATa, DH, KR, ACP; module 4: KS, ATa, DH, KR, ACP)	*S. avermitilis* MA-4680	48/59
*abyB2*	31 269	3645	PKS I (module 5: KS, ATp, DH, KR, ACP; module 6: KS, ATp, DH, ER, KR, ACP)	*Streptomyces* sp. DSM 21069	50/61
*abyB3*	42 203	992	PKS I (module 7: KS, ATa, ACP)	*S. antibioticus*	54/65
*abyC*	45 942	230	regulatory protein, TetR	*Frankia* sp. EAN1pec	60/75
				*Brucella abortus biovar* 1 str. 9-941	40/56
*abyD*	46 026	475	drug resistance transporter EmrB/QacA subfamily	*Frankia* sp. EAN1pec	66/79
			export protein	*S. antibioticus*	44/60
*abyE*	47 524	335	luciferase; alkanal monooxygenase α-chain	*Frankia* sp. EAN1pec	60/74
			flavin-utilizing monooxygenases	*Brucella melitensis* 16M	45/64
*abyF1*	48 619	538	ABC transporter oligopeptide binding protein	*Frankia alni* ACN14a	45/59
			peptide ABC transporter, periplasmic peptide-binding protein	*Klebsiella pneumoniae* 342	40/55
*abyF2*	50 232	311	ABC transporter oligopeptide permease	*Frankia alni* ACN14a	53/74
			binding protein-dependent transport systems inner membrane component	*Frankia* sp. CcI3	51/69
*abyF3*	51 164	283	binding protein-dependent transport systems inner membrane component	*Frankia* sp. EAN1pec	54/70
			oligopeptide ABC transporter permease protein	*Symbiobacterium thermophilum* IAM 14863	45/62
*abyF4*	52 005	539	peptide ABC transporter ATP-binding protein	*Frankia alni* ACN14a	59/71
			ABC transporter ATP-binding protein	*Janthinobacterium* sp. *Marseille*	52/63
*abyV*	53 621	395	cytochrome P450	*Frankia* sp. EAN1pec	63/72
			cytochrome P450 hydroxylase	*S. avermitilis* MA-4680	51/63
*abyW*	54 771	302	alcohol dehydrogenase zinc-binding domain protein	*S. bingchenggensis* BCW-1	64/76
			oxidoreductase	*Streptomyces hygroscopicus* ATCC 53653	60/74
*abyZ*	55 601	165	NAD(P)H-dependent FMN reductase	*S. clavuligerus* ATCC 27064	73/81
				*S. viridochromogenes* DSM 40736	75/86
*abyT*	55 726	298	thioesterase	*Nostoc punctiforme* PCC 73102	35/52
			oleoyl-(acyl-carrier protein) hydrolase	*Haliangium ochraceum* DSM 14365	42/55
*orfU*	57 429	302	alcohol dehydrogenase zinc-binding domain protein	*S. bingchenggensis* BCW-1	64/76
			oxidoreductase	*S. hygroscopicus* ATCC 53653	60/74
*orfS*	57 520	197	transcriptional regulator, TetR family protein	*S. bingchenggensis* BCW-1	59/70
			putative transcriptional regulator	*S. hygroscopicus* ATCC 53653	57/72

Upstream of *abyA1*–*A5*, five genes were considered to be part of the *aby* cluster: *abyH*, a LuxR transcriptional regulator homologue; *abyI and abyR*, genes encoding putative pathway specific activator proteins (SARP family); *abyX*, a cytochrome P450 gene and *abyK* encoding a protein with YD repeats. Gene *orfQ*, encoding a putative NUDIX hydrolase, is not considered to be necessary for abyssomicin C biosynthesis. Downstream of the PKS I genes, those encoding a TetR-like regulatory protein (*abyC*), a drug resistance transporter belonging to the EmrB/QacA subfamily (*abyD*), a monooxygenase (*abyE*), an ABC-transporter system (*abyF1–F4*), a cytochrome P450 system *(abyV*, *abyW* and *abyZ*) and a type II thioesterase (*abyT*) form part of the putative gene cluster, whereas *orfU*, encoding an alcohol dehydrogenase-like protein, and *orfS*, a TetR-like regulatory protein, were not considered to be involved in abyssomicin formation. Finally, the identity of the gene cluster was confirmed by inactivation of PKS I in *abyB1* by single crossover integration by using a fragment internal to the gene cloned into the nonreplicating vector pK18mob2; this abolished *atrop*-abyssomicin C production as judged by LC-ESI-MS analysis.

Three genes in the *aby* cluster code for a seven-module type I PKS assembly line for the polyketide backbone of abyssomicin C (Scheme 2). The first gene, *abyB1*, consists of four modules with the minimal set of ketosynthase (KS), acyltransferase (AT) and acyl carrier protein (ACP) for the first module. The subsequent three modules consist of the same basic set, each extended by dehydratase (DH) and ketoreductase (KR) domains arranged collinearly with their functions in the biosynthetic assembly line. Module 5 of *abyB2* has the same arrangement as module 4; the presence of a DH and KR would appear to contradict the collinearity rule.^16^ However, consistent with the proposed model of biosynthesis (Scheme 2) the DH and KR domains of module 5 (AbyB2–DH1 and AbyB2–KR1, respectively) are likely to be inactive. The active site for DH domains is a His–Asp catalytic dyad,^17^ while an active site triad of Lys, Ser and Tyr residues is required for KR activity.^18^ Using AbyB2 numbering, AbyB2–DH1 has substitutions His→Leu953 and Asp→Thr1184, while AbyB2–KR1 has Lys→Ile1402 and Tyr→Val1439. Module 6 contains an additional enoylreductase (ER) domain while module 7 of AbyB3 adds an additional acetate unit. The specificity of AbyB2–AT1 and AbyB2–AT2 for propionate incorporation is determined by the amino acids following Arg56: RVDVV-7M-1-S-1-AXhW (the motif described by Haydock et al.^18^). The published consensus sequence for acetate specificity of the rest of the AT domains is less well conserved in the *abyC* cluster (corresponding conserved residues are shown in bold): instead of E**T**GY**A**-7-**Q**-1**A**-1-**F**G**LL**,^18^ R**T**E-1-**AQ**P**A**l**F**a-1-E-1-AL-2-**LL** is found in a sequence alignment of all acetate-carrying AT domains.

**Scheme 2:**
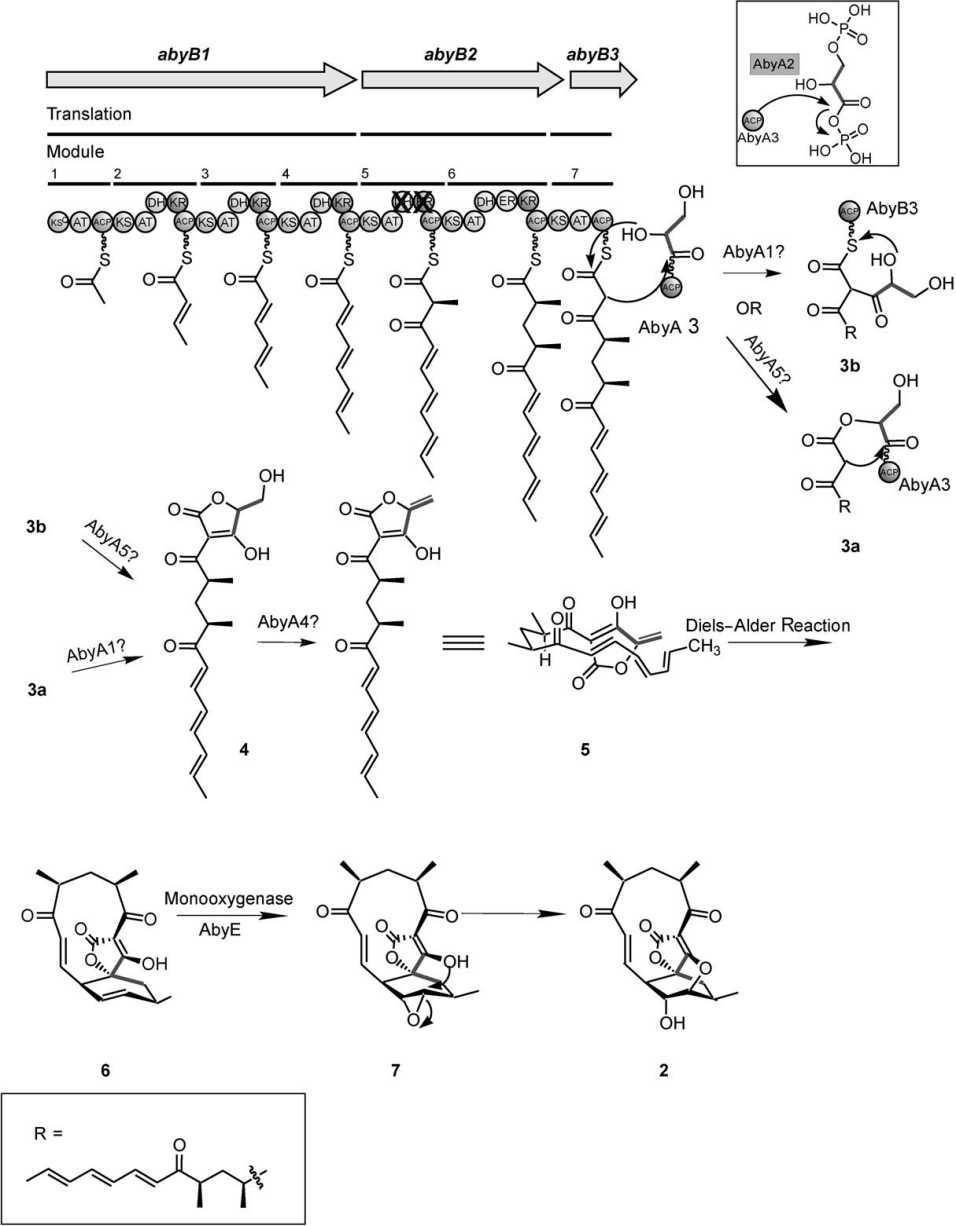
Model of the *atrop*-abyssomicin C biosynthesis shows the formation of a linear precursor from acetate and propionate precursors on AbyB1, AbyB2 and AbyB3. Bisphosphoglycerate from primary metabolism is attached to the discrete ACP, AbyA3, by the acyl transferase, AbyA2. Subsequently, transfer of the linear polyketide on AbyA3 carrying glyceraldehyde is followed by the formation of the tetronate unit **4**. The order of reactions for the C–C and C–O bonds remains undefined. AbyA1 is similar to a β-ketoacyl-ACP-synthase III, which is known to catalyse the first condensation step in fatty acid biosynthesis, and could catalyse formation of the C–C bond to yield derivative **3 b** and finally **4**.12a AbyA5 shows homology to the α/β-hydrolase protein family and could catalyse the C–O bond and lead to derivative **3 a** and finally **4**. In other tetronic acid antibiotic biosynthetic gene clusters, an FAD-dependent oxidoreductase has been proposed to catalyse the dehydration of **4** to yield **5**.12a, d However, in the *atrop*-abyssomicin C biosynthetic gene cluster, this gene is not present. Instead, AbyA4, a dehydrogenase catalytic domain-containing protein could catalyse this reaction. Following the formation of the tetronic acid moiety, a Diels–Alder reaction is proposed to form product **6**. After suggested epoxide formation by the oxygenase AbyE, ring opening of **7** leads to formation of *atrop*-abyssomicin C (**2**).

All four previously sequenced tetronate biosynthetic gene clusters (chlorothricin: *chl*;12c kijanimicin: *kij*;12d tetronomycin: *tmn*;12a tetrocarcin: *tca*12b) contain a unique set of four or five genes. In the case of tetrocarcin and kijanimicin, two genes (homologues of *chlD3* and *clhD4*) are fused together to form a single bifunctional enzyme (TcaD3 and KijE, respectively).12b Originally, it was proposed that only four of these genes are involved in tetronic acid biosynthesis since inactivation of *chlM* of the chlorothricin gene cluster did not abolish chlorothricin production. Recent elucidation of the biosynthetic gene clusters of three more tetronic acid-containing antibiotics, and the identification of additional copies of *chlM* in the genome of the chlorothricin producer (which would explain why inactivation of *chlM* in the *chl* cluster did not interrupt chlorothricin biosynthesis) suggests that all five conserved genes play a role.12a, b, d The presence of homologues of these five genes (*abyA1–A5*) in the abyssomicin gene cluster strengthens this hypothesis (see Scheme 2 for a proposed biosynthetic pathway).

In this study, in-frame deletions of *abyA1*, *abyA2*, *abyA3* and *abyA4* completely abolished abyssomicin production; this confirms their involvement in *atrop*-abyssomicin C biosynthesis (Scheme 2). However, biochemical evidence for the individual steps in tetronic acid biosynthesis remains scarce and only the first two steps have been examined in vitro.12a Firstly, a glyceryl moiety from d-1,3-bisphosphoglycerate is transferred to Tmn7a and ChlD2 (discrete ACPs homologous to AbyA3), by Tmn16 (a homologue of FkbH and AbyA2), leading to glyceryl-*S*-ACP (Scheme 2). The second step, that is, the binding of glyceryl-*S*-ACP to the nascent polyketide chain and detachment of the polyketide from the polyketide synthase, remains elusive together with the subsequent dehydration. Sun et al.^19^ used a “minimal set” of only six genes and suggested that *rkD*, a 3oxoacyl-acyl carrier protein synthase III (homologue of *abyA1*), is capable of catalysing both C–C and C–O bond formation during biosynthesis of the tetronate moiety.12a

However, it remains to be seen whether these results can be directly transferred to the more complex biosynthetic pathways for tetronates, such as abyssomicin, tetronomycin or tetrocarcin. Further experiments will be needed to determine the precise mechanisms involved. For initiation of tetronate ring formation of abyssomicin, we propose the C–O bond as the first step (Scheme 2). This is performed by attack of the primary hydroxyl group of the glyceryl-ACP (AbyA3) on the polyketide thioester on AbyB3. This reaction might be catalysed by an acyltransferase/thioesterase (AbyA5). Formation of the C–O bond would release the polyketide from the polyketide synthase enzyme, AbyB3. Subsequent formation of the tetronate ring could occur by nucleophilic attack of the C-2 carbon. This step could be catalysed by AbyA1 but also an uncatalysed reaction from the enol-form is conceivable. Alternatively, AbyA5, ChlD4 and Tmn17, as predicted acyltransferases, could catalyse this C–O bond formation step.12a

Interestingly, a gene encoding a FAD-dependent oxidoreductase like KijA or Tmn9, which has been suggested to be involved in the formation of the tetronic acid moiety by catalysing dehydration and subsequent Diels–Alder reaction, was not found in the abyssomicin gene cluster. For the chlorothricin and tetrocarcin gene clusters, cross complementation experiments, in vivo, have shown that the homologues of AbyA3 and AbyA4 (encoding an ACP and dehydrogenase, respectively) are interchangeable, which is not surprising given the strong structural similarity between chlorothricin and tetronomycin.12b Sequence comparison of the five tetronic acid biosynthetic gene clusters at the protein level revealed that kijanimicin and tetrocarcin are very closely related (78 % overall identity), followed by chlorothricin (63 % identity with kijanimicin). *Atrop*-abyssomicin and tetronomycin show 61 % amino acid sequence identity (see the Supporting Information, phylogenetic analysis and percentage identity table).

After formation of the tetronic acid moiety to give linear intermediate **5** (Scheme 2), we propose the occurrence of an intramolecular Diels–Alder reaction to yield intermediate **6**. Many natural products are postulated to be Diels–Alder products.^20^ However, so far only two natural enzymes have been purified to homogeneity and their role as catalysts for Diels–Alder reactions confirmed.^21^ Analysis of the *aby* gene cluster did not identify a candidate for such an enzyme, neither did any of the gene-inactivation experiments yield a linear precursor. From work on the total synthesis of abyssomicin, it is known that the methoxy-protected analogue of the linear tetronate is converted to the corresponding Diels–Alder product under very mild conditions:9c after one week at 25 °C in CDCl_3_, 40 % had reacted to form the methoxy-protected analogue of **6**. Hence, it is reasonable to assume that *atrop*-abyssomicin C can be formed under physiological conditions in the absence of a putative Diels–Alderase. Likewise, the gene clusters for other members of the tetronate family, kijanimicin, chlorothricin and tetrocarcin, for which Diels–Alder reactions have also been proposed,12c, d do not suggest enzyme candidates for a Diels–Alderase. The only candidate proteins for supporting or execution of Diels–Alder reaction are the polyketide synthases (AbyB1–3) and proteins involved in post-PKS modifications.

The biosynthetic logic of abyssomicin assembly further requires the formation of epoxide **7** (Scheme 2), which can then be opened by the tetronate hydroxyl group. AbyE is a putative monooxygenase, which is highly homologous to a large family of bacterial luciferase-like proteins. Little is known about this family and most members are not biochemically characterised. InterProScan^22^ analysis shows that bacterial luciferases are flavin monooxygenases that catalyse the oxidation of long-chain aldehydes and release energy in the form of visible light by using flavin as a substrate rather than a cofactor. In-frame deletion of *abyE* led to a significant reduction of *atrop*-abyssomicin C production (less than 10 % of wild-type levels). Detailed analysis of the culture filtrate and extract by using HPLC-ESI-MS showed no candidate masses for possible intermediary structures.

AbyX is a cytochrome P450, and thus, also a candidate for oxygenation of Diels–Alder product **6** (Scheme 2). In-frame deletion of *abyX* led to reduction of abyssomicin production to about 3 % of wild-type levels. One further candidate for oxygenation of **6** is cytochrome P450, AbyV. Interruption of *abyV* with single crossover recombination led to loss of abyssomicin production; however, polar effects on the expression of downstream genes cannot be ruled out. Detailed analysis of the culture filtrates of both mutants by using HPLC-MS did not show any candidate masses for possible intermediary structures. Failure to detect any biosynthetic intermediate by LC-ESI-MS prior to oxidative epoxide formation was unexpected, since precursors **5** and **6** were expected to be released by the polyketide assembly line, and mass spectrometry is the method of choice even if the compounds are unstable. Ultimately, in vitro experiments will be needed to determine the exact mechanism for oxygenation of the proposed Diels–Alder product **6**.

Upstream of the PKS I genes, there are three genes encoding homologues of known regulators of secondary metabolism: *abyH*, encoding a LuxR family transcriptional regulator, and *abyI* and *abyR*, both encoding putative pathway specific activator proteins belonging to the SARP (*Streptomyces* antibiotic regulatory protein) family of transcriptional activators. As expected, in-frame deletion of *abyI* abolished abyssomicin production. Complementation of the mutant with a copy of the gene by using vectors pSET*ermE*ΔHindIII and pUWL*oriT* restored production to wild-type levels in both cases (for details see the Supporting Information). In-frame deletion of *abyR* led to a reduction in abyssomicin production; this suggests some degree of involvement in regulation of abyssomicin biosynthesis.

LuxR regulators, like AbyH can be activators or repressors, which, acting through a quorum sensing mechanism, induce transcription when a certain cell density is reached.^23^ The role of AbyH in abyssomicin production remains to be determined.

According to its sequence data AbyD belongs to the major facilitator superfamily (MFS) of bacterial transporters. MFS transporters are capable of transporting small solutes in response to chemiosmotic ion gradients.^24^ AbyD belongs to the EmrB/QacA subfamily of drug efflux pumps, related to the tetracycline resistance protein, TetB. Many multidrug transporters in this superfamily are negatively regulated by the TetR family of transcriptional regulators.^25^ Consistent with this is the location of *abyC* (predicted to encode a TetR-like transcriptional regulator), which is upstream of, and divergently transcribed from, *aby*D. Examination of the intergenic region between *abyC* and *abyD* revealed a 16 bp perfect palindromic repeat sequence, TGAACTGATCAGTTCA, that is likely to be the operator binding sequence for AbyC. In-frame deletions of *abyC* and *abyD* both resulted in a reduction of *atrop*-abyssomicin C production to less than 10 % of the wild-type level. Self-resistance might, therefore, stem from the efficient export of abyssomicin from the cell; the low level of abyssomicin production observed in the Δ*abyC* and Δ*abyD* strains can be explained by a degree of passive diffusion from the cell or by partial suppression of Δ*abyD* by another MFS transporter with broader substrate tolerance. The former possibility has been suggested to explain the excretion of heterologously produced polyfunctional aromatic compounds by mutants of *Streptomyces coelicolor* that lack the actinorhodin transporters.^26^

Downstream of *abyD* are *abyF1–4*, which are predicted to encode an ABC transporter system. Mutations in these genes have yet to be made. In all cases studied so far, ABC transporters that contain an extracellular solute binding protein, such as that encoded by AbyF1, are importers—although sometimes efflux is also facilitated, it always leads to a net uptake of solutes.^27^ This suggests that these genes might be involved in the biosynthesis, rather than export of *atrop*-abyssomicin C, and possibly in the import of a compound required for *atrop*-abyssomicin C production. While the precursors for biosynthesis should be present in the intracellular metabolite pool, *Verrucosispora maris* AB-18-032 requires the presence of glycerol in the fermentation medium for *atrop*-abyssomicin C production. Glycerol is not the origin of the three-carbon unit incorporated into the C-14–C-16 position of *atrop*-abyssomicin C, but it functions as an autoinducer of pimaricin biosynthesis in *Streptomyces natalensis* and stimulates the production of six different macrolide antibiotics.^28^ Glycerol is imported by an ABC transporter complex in *Mycobacterium* species,^27^ and thus one possible role for AbyF1–4 is the uptake of glycerol or another solute involved in stimulating *atrop*-abyssomicin C production.

For many of the *aby* genes, the highest similarity is to a counterpart in *Frankia alni* ACN14a and/or *Frankia* sp. EAN1pec, both found in whole genome sequencing projects.^29^ Hence, an interesting profile of secondary metabolite production can be expected from these organisms. Examination of the genome of *Frankia alni* ACN14a (GenBank: CT573213.2) revealed a putative spirotetronate biosynthetic gene cluster that contains homologues of most of the genes in the *aby* cluster, including *abyK* (Figure 2). This region (FRAAL4069–4097) contains *abyF1–4* homologues in proximity to those for a MFS protein, a TetR regulator, a monooxygenase and a cytochrome P450. Elsewhere in the genome of *Frankia alni* (ddpD-FRAAL0342) this gene organisation is repeated with greater synteny to the *aby* cluster, but the product of the biosynthetic gene cluster in this region is not predicted to be a tetronate as it lacks homologues of the genes required for the biosynthesis of the tetronic acid moiety (see above).

**Figure 2:**
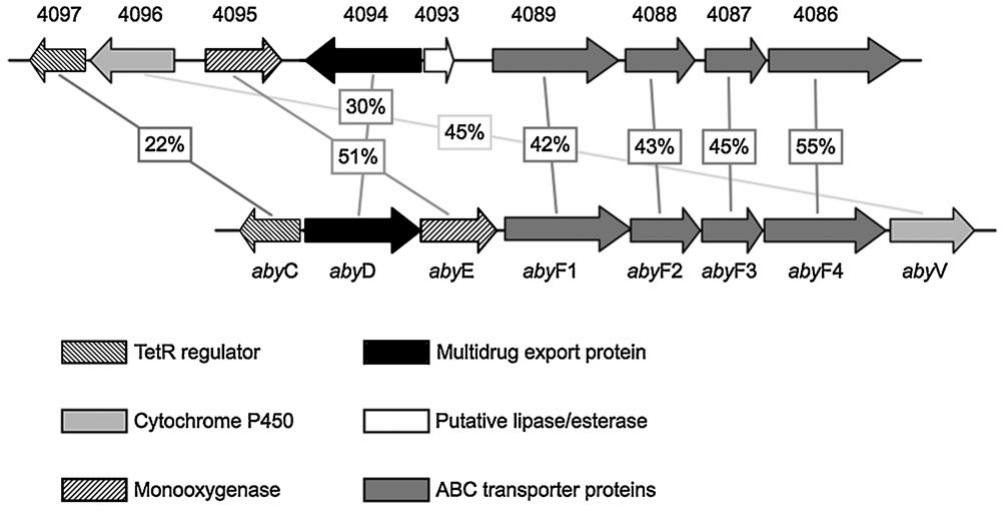
Comparison of the genes predicted to be involved in the export of abyssomicin and a putative spirotetronate biosynthetic gene cluster present in the genome of *Frankia alni* ACN14a (GenBank accession: CT573213.2). The numbers in boxes represent the percentage identify (amino acid) between the proteins (joined by lines). The putative lipase/esterase might function as an editing thioesterase as predicted for *abyT* (located downstream of *abyV*).

According to InterProScan analysis, *abyK* contains several tandem copies of a 21 residue extracellular repeat that is found in Gram-negative, Gram-positive, and animal proteins. The repeat is named for a YD dipeptide, the most strongly conserved motif of the repeat. These repeats appear to be involved in binding carbohydrates; for example, the chicken teneurin-1 YD-repeat region binds heparin.^30^ In-frame deletion of *abyK* led to a decrease of abyssomicin C production to roughly 30 % of wild-type levels. Complementation of the knockout mutant with a copy of the gene on a plasmid transcribed from the ermE* promoter restored production to wild-type levels. Interestingly, homologues of this gene are contained in the gene clusters for tetrocarcin, chlorothricin and tetronomycin, albeit as shorter versions (164, 191 and 190 aa, respectively). In the gene clusters for tetrocarcin and tetronomycin, the YD-repeat proteins are located directly upstream of the genes responsible for tetronic acid biosynthesis. Therefore, it is likely that this gene has some relevance for the biosynthesis of the aforementioned tetronates, however, its exact function remains elusive.

Discrete type II thioesterase domains (TEs) have been predicted to play a role in editing stalled polyketide chains.^26^ The TEs are thought to patrol the PKS assembly line in trans, and hydrolytically remove any inappropriate monomers misincorporated by the AT domains.^31^ The chlorothricin, kijanimicin, tetrocarcin and tetronomycin biosynthetic gene clusters all have discrete TEs (ChlK, KijB2, TcaF and Tmn6, respectively). In-frame deletion of *abyT* resulted in approximately 50 % lower levels of production than that of the wild type, which is consistent with an editing function in abyssomicin biosynthesis.

Spirotetronates form a highly complex, and very often, extremely functionalised class of polyketides. Among these, *atrop*-abyssomicin C is the smallest and simplest example of a spirotetronate, the biosynthetic gene cluster of which has been determined. Apart from the comparatively short polyketide chain (seven modules) we found five genes (*abyA1*–*abyA5*) assigned to enzymes catalysing formation of the tetronic acid moiety and three genes coding for oxidising enzymes (*abypE*, *abyX* and *abyV*). Apart from one gene, the function of which remains obscure (*abyK*), the remaining genes of the cluster are assigned to export, possibly import, and regulatory functions (*abyI*, *abyH*, *abyC*, *abyD*, *abyR*, *abyI*, *abyT* and *abyF1*–*abyF4*). The work reported here describes the first example of the genetic manipulation of a member of the genus *Verrucosispora*, and paves the way for future detailed studies of secondary metabolism in this group of scarcely studied microorganisms.

## Experimental Section

**Fermentation and isolation:** For growth of *Verrucosispora maris* AB-18-032 strains, the production medium SGG (1 % dextrin, 1 % glucose, 1 % glycerol, 0.25 % corn steep powder from Marcor, Carlstadt, NJ, USA, 0.5 % peptone, 0.2 % yeast extract, 0.1 % NaCl, 0.3 % CaCO_3_ in 1 L tap water, adjusted to pH 7.3 prior to sterilisation) was dispersed in Erlenmeyer flasks (500 mL; 10× for each cultivation) with three baffles. The flasks were inoculated with 3 *v* % of a 48 h old preculture and incubated at 27 °C and 120 rpm on a rotary shaker. After 72 h the cultures were harvested by centrifugation. The supernatants were used for isolation of abyssomicins, as described previously.3a

^**13**^**C-Isotope labelling studies:** In parallel experiments 500 mg of ^13^C-labelled precursors [1-^13^C]acetate, [1,2-^13^C]acetate and [1-^13^C]propionate, and [1,2-^13^C]glucose (250 mg; Eurisotop, Saint Aubin, France) were fed to producing cultures (1 L) of *Verrucosispora maris* AB-18-032 in SGG liquid medium in five portions: at 48, 51, 54, 57 and 60 h after inoculation. After 72 h of cultivation, the bacteria were harvested and ^13^C-labelled *atrop*-abyssomicins C was isolated.3a

**HPLC-ESI-mass spectrometry:** The supernatants of 72 h cultures (5 mL) of *Verrucosispora maris* AB-18-032 were adjusted to pH 4 (aq. HCl) and subsequently extracted with ethyl acetate (5 mL). The organic phase was separated and the solvent evaporated, in vacuo. The dry residue was dissolved in methanol (500 μL), centrifuged and analysed on an Agilent 1100 analytical HPLC system (Waldbronn, Germany) equipped with a Luna RP-C18 column (4.6 mm i.d., Phenomenex, Aschaffenburg, Germany) and a diode array detector (5 % B (acetonitrile) to 100 % B in 10 min, flow rate: 1.5 mL min^−1^, *t*_R_=5.6 min). Relative production was estimated by peak areas compared to wild-type fermentations. ESI-MS measurements: single-ion monitoring set to *m/z* 374.1, polarity: positive, fragmentor: 135 V (ESI-Triple-Quadrupol-MS, 6460 Series, Agilent Technologies).

**NMR spectroscopy:** ^1^H- and ^13^C NMR spectroscopy experiments were measured on a DRX500 NMR spectrometer (Bruker) equipped with a 5 mm diameter broad band inverse probe head with *z* gradients. Spectra were recorded in and referenced to [D_4_]methanol (3.30 ppm; 49.0 ppm). Additional spectra are shown in the Supporting Information.

**Bacterial strains, plasmids, and reagents:** *E. coli* strains and plasmids used in this work were obtained from Combinature Biopharm, AG (Berlin, Germany). Biochemicals, chemicals and media were obtained from Roth unless indicated otherwise. Restriction enzymes and molecular biological reagents were obtained from standard commercial sources, such as Qiagen, New England Biolabs and Fermentas. Primers were ordered from biomers.net (Ulm, Germany).

**DNA isolation, genomic library construction and screening:** A cosmid library of *Verrucosispora maris* AB-18-032 genomic DNA (2304 clones) was constructed, as described previously.^32^ Homogenised bacterial cultures were immersed in low-melting agarose (0.5 %; SeaPlaque GTG, Biozym, Hessisch Oldendorf, Germany) and incubated with HEW lysozyme (2 mg mL^−1^; Roth) for 14 h at room temperature and with proteinase K (1 mg mL^−1^; Merck) for 24 h at 50 °C. The embedded DNA was partially digested with Sau3AI and extracted with Gelase (Epicentre, Madison, WI, USA) and dephosphorylated (Antarctic Phosphatase, New England Biolabs). The fragments of genomic DNA (∼50 kb long) were ligated with BamHI digested vector pOJ436 (750 ng), desalted and packaged by using Gigapack III Gold packaging extract (Stratagene).^33^ The cosmid clones were screened by hybridisation with a DIG-labelled PKS probe (Roche DIG-labelling kit), which was amplified from *Verrucosispora maris* AB-18-032 genomic DNA by using primers KSIIFOR (5′-CTSGGSGACCCSATCGAG-3′) and ATIREV (5′-GCSGCSGCGATCTCSCCCTGSSWGTGSCC-3′); 49 PKS I positive cosmid clones were identified.

**Whole genome sequencing (WGS) of** ***Verrucosispora maris*** **AB-18-032:** Genomic DNA from *Verrucosispora maris* AB-18-032 was prepared by using a standard CTAB procedure.^33^ Genomic DNA was further purified by passaging twice through a 20/G genomic-tip according to the manufacturers instructions (Qiagen). Purified DNA was used for whole-genome shotgun pyrosequencing by using a Genome Sequencer 20 instrument (Roche) and GS emPCR Kit I, according to the manufacturers instructions (Roche). Pryosequencing reads representing 130 Mb of data (ca. 20.4× coverage) were assembled by using the Newbler assembler version 1.1.03.24 (Roche) into 1276 contigs with an N50 contig size of 8455 bp.

**Cosmid analysis and shotgun cloning:** To identify cosmids carrying PKS I genes for abyssomicin C biosynthesis, all 49 cosmids containing PKS I genes were digested with BamHI and SmaI and separated on an agarose gel. After hybridisation with DIG-labelled PKS I probes, cosmids were placed into families containing overlapping genomic sequences. Three major families (five to eight members each) and four smaller families (two members each) as well as 14 apparently unique cosmids were identified. One cosmid from each family was chosen and subjected to restriction digestion with BamHI. The fragments were cloned in pBKS and sequenced. PKS I positive inserts were then cloned into pK18mob2^34^ and used for mutational inactivation in *Verrucosispora maris* AB-18-032 by single crossover recombination. The resulting mutants were screened for the absence of abyssomicin C production. Cosmid family II was found to carry genes required for abyssomicin C biosynthesis and one cosmid from this family (C17) was chosen for sequencing.

**Cosmid sequencing and sequence assembly:** DNA sequencing was carried out by AGOWA GmbH (Berlin, Germany). Cosmid C17 (insert size 35 567 bp) was sequenced by shotgun sequencing (4× coverage); overlapping regions on cosmids C45 and C49 were identified and sequenced by chromosome walking.

**Sequence analysis:** Open reading frame (ORF) prediction was carried out by using Glimmer (http://cbcb.umd.edu/software/glimmer/) and Frameplot (http://www.nih.go.jp/∼jun/cgi-bin/frameplot.pl). Protein functions were assigned with an xblast (http://www.ncbi.nlm.nih.gov/blast/) search as well as an InterProScan (http://www.ebi.ac.uk/InterProScan/) domain analysis. WGS contigs containing DNA from the *aby* gene cluster were identified by BLAST searching, and were manually annotated by using Artemis sequence visualisation and annotation software^35^ and BLAST search tools. The annotated sequence is available at http://www.ncbi.nlm.nih.gov/Genbank/index.html under GenBank accession number JF752342.

**Gene inactivation:** Two different methods of gene inactivation were applied. Function assignment and cluster boundary assignment was achieved with single crossover homologous recombination by using plasmid pK18mob2.^34^ In-frame deletion of genes was achieved following the ReDirect protocol by using λ-Red-mediated recombination, and classical gene deletion methods.^36^ Procedures, primers and constructs are described in the Supporting Information.

## Supplementary material

Detailed facts of importance to specialist readers are published as ”Supporting Information”. Such documents are peer-reviewed, but not copy-edited or typeset. They are made available as submitted by the authors.

## References

[1a] Fiedler HP, Bruntner C, Bull AT, Ward AC, Goodfellow M, Potterat O, Puder C, Mihm G (2005). Antonie van Leeuwenhoek.

[1b] Herold K, Gollmick FA, Groth I, Roth M, Menzel KD, Mollmann U, Grafe U, Hertweck C (2005). Chem. Eur. J.

[1c] Takahashi Y, Omura S (2003). J. Gen. Appl. Microbiol.

[1d] Tsurumi Y (2003). Kagaku (Kyoto, Japan).

[2] Clardy J, Fischbach MA, Walsh CT (2006). Nat. Biotechnol.

[3a] Bister B, Bischoff D, Ströbele M, Riedlinger J, Reicke A, Wolter F, Bull AT, Zähner H, Fiedler HP, Süssmuth RD (2004). Angew. Chem.

[b37] (2004). Angew. Chem. Int. Ed.

[3b] von Nussbaum F, Brands M, Hinzen B, Weigand S, Häbich D (2006). Angew. Chem.

[b38] (2006). Angew. Chem. Int. Ed.

[4] Coggins CAJR, Evans LB, Frederickson M, Robinson DA, Roszak AW, Lapthorn AP (2003). Biochem. Soc. Trans.

[5a] Domagk G (1935). Dtsch. Med. Wochenschr.

[5b] Fuller AT (1937). Lancet.

[5c] Northey EH (1940). Chem. Rev.

[5d] Nicol CA, Welch AD (1950). Proc. Soc. Exp. Biol. Med.

[5e] Burchall GHHJ (1965). Mol. Pharmacol.

[6] Riedlinger J, Reicke A, Zähner H, Krismer B, Bull AT, Maldonado LA, Ward AC, Goodfellow M, Bister B, Bischoff D, Süssmuth RD, Fiedler HP (2004). J. Antibiot.

[7] Keller S, Nicholson G, Drahl C, Sorensen E, Fiedler HP, Süssmuth RD (2007). J. Antibiot.

[8a] Schadt HS, Schadt S, Oldach F, Süssmuth RD (2009). J. Am. Chem. Soc.

[8b] Keller S, Schadt H, Ortel I, Süssmuth RD (2007). Angew. Chem.

[b39] (2007). Angew. Chem. Int. Ed.

[9a] Nicolaou KC, Harrison ST (2006). Angew. Chem.

[b40] (2006). Angew. Chem. Int. Ed.

[9b] Peters R, Fischer DF (2006). Angew. Chem.

[b41] (2006). Angew. Chem. Int. Ed.

[9c] Snider BB, Zou Y (2005). Org. Lett.

[9d] Zapf CW, Harrison BA, Drahl C, Sorensen EJ (2005). Angew. Chem.

[b42] (2005). Angew. Chem. Int. Ed.

[9e] Zhang XJ, Parry RJ (2007). Antimicrob. Agents Chemother.

[9f] Rath J-P, Eipert M, Kinast S, Maier ME (2005). Synlett.

[9g] Rath J-P, Kinast S, Maier ME (2005). Org. Lett.

[10] Nicolaou KC, Harrison ST (2007). J. Am. Chem. Soc.

[11] Schindler PW, Zähner H (1973). Eur. J. Biochem.

[12a] Demydchuk Y, Sun YH, Hong H, Staunton J, Spencer JB, Leadlay PF (2008). ChemBioChem.

[12b] Fang J, Zhang Y, Huang L, Jia X, Zhang Q, Zhang X, Tang G, Liu W (2008). J. Bacteriol.

[12c] Jia XY, Tian ZH, Shao L, Qu XD, Zhao QF, Tang J, Tang GL, Liu W (2006). Chem. Biol.

[12d] Zhang H, White-Phillip JA, Melancon CE, Kwon HJ, Yu WL, Liu HW (2007). J. Am. Chem. Soc.

[13a] Chijiwa HRPS, Furihata K, Ogata M, Endo T, Kuzuyama T, Hayakawa Y, Shin-Ya K (2003). Tetrahedron Lett.

[13b] Lee JPLJJ, Keller PJ, Cottrell CE, Chang CJ, Zähner H, Floss HG (1986). J. Antibiot.

[13c] Mashimo Y, Sekiyama Y, Araya H, Fujimoto Y (2004). Bioorg. Med. Chem. Lett.

[13d] Tamaoki T, Tomita F (1983). J. Antibiot.

[14a] Byrne KM, Arison BH, Nallinomstead M, Kaplan L (1993). J. Org. Chem.

[14b] Michal G (1999). Biochemical Pathways: Biochemie-Atlas.

[15a] Weber T, Welzel K, Pelzer S, Vente A, Wohlleben W (2003). J. Biotechnol.

[15b] Weber KJLT, Pross EK, Textor A, Grond S, Welzel K, Pelzer S, Vente A, Wohlleben W (2008). Chem. Biol.

[16] Donadio S, Katz L (1992). Gene.

[17] Akey JRRDL, Tehranisa J, Sherman DH, Gerwick WH, Smith JL (2010). Structure.

[18] Haydock SF, Aparicio JF, Molnar I, Schwecke T, Khaw LE, Konig A, Marsden AFA, Galloway IS, Staunton J, Leadlay PF (1995). FEBS Lett.

[19] Sun FHY, Demydchuk Y, Chettle J, Tosin M, Osada H, Leadlay PF (2010). Nat. Chem. Biol.

[20] Stocking EM, Williams RM (2003). Angew. Chem.

[b43] (2003). Angew. Chem. Int. Ed.

[21] Kelly WL (2008). Org. Biomol. Chem.

[22] Zdobnov EM, Apweiler R (2001). Bioinformatics.

[23] Nasser W, Reverchon S (2007). Anal. Bioanal. Chem.

[24] Pao SS, Paulsen IT, Saier MH (1998). Microbiol. Mol. Biol. Rev.

[25] Ramos JL, Martinez-Bueno M, Molina-Henares AJ, Teran W, Watanabe K, Zhang X, Gallegos MT, Brennan R, Tobes R (2005). Microbiol. Mol. Biol. Rev.

[26] Fischbach MA, Walsh CT (2006). Chem. Rev.

[27] Vilei EM, Frey J (2001). Clin. Diagn. Lab. Immunol.

[28] Recio E, Aparicio JF, Rumbero A, Martin JF (2006). Microbiology.

[29] Normand P, Lapierre P, Tisa LS, Gogarten JP, Alloisio N, Bagnarol E, Bassi CA, Berry AM, Bickhart DM, Choisne N, Couloux A, Cournoyer B, Cruveiller S, Daubin V, Demange N, Francino MP, Goltsman E, Huang Y, Kopp OR, Labarre L, Lapidus A, Lavire C, Marechal J, Martinez M, Mastronunzio JE, Mullin BC, Niemann J, Pujic P, Rawnsley T, Rouy Z, Schenowitz C, Sellstedt A, Tavares F, Tomkins JP, Vallenet D, Valverde C, Wall LG, Wang Y, Medigue C, Benson DR (2007). Genome Res.

[30a] Hill CW, Sandt CH, Vlazny DA (1994). Mol. Microbiol.

[30b] Feulner G, Gray JA, Kirschman JA, Lehner AF, Sadosky AB, Vlazny DA, Zhang J, Zhao S, Hill CW (1990). J. Bacteriol.

[30c] Minet A, Rubin B, Tucker R, Baumgartner S, Chiquet-Ehrismann R (1999). J. Cell Sci.

[31] Kim BS, Cropp TA, Beck BJ, Sherman DH, Reynolds KA (2002). J. Biol. Chem.

[32a] Burgtorf C, Welzel K, Hasenbank R, Zehetner G, Weis S, Lehrac H (1998). Genomics.

[32b] Beye M, Poch A, Burgtorf C, Moritz RFA, Lehrach H (1998). Genomics.

[33] Kieser T, Bibb MJ, Buttner MJ, Chater KF, Hopwood DA (2000). Practical Streptomyces Genetics.

[35] Carver T, Berriman M, Tivey A, Patel C, Böhme U, Barrell BG, Parkhill J, Rajandream M-A (2008). Bioinformatics.

[36a] Gust B, Challis GL, Fowler K, Kieser T, Chater KF (2003). Proc. Natl. Acad. Sci. USA.

[36b] Gust B, Chandra G, Jakimowicz D, Tian YQ, Bruton CJ, Chater KF (2004). Adv. Appl. Microbiol.

