# Supplementary material for: Abyssomicin Biosynthesis: Formation of an Unusual Polyketide, Antibiotic-Feeding Studies and Genetic Analysis
Source: Chembiochem. 2011 Jun 9;12(9):1401–10. doi: 10.1002/cbic.201100172 (PMC3625739; doi:10.1002/cbic.201100172)
Supplement: Supplementary file 1 [file cbic0012-1401-SD1.pdf]

CHEMBIOCHEM

Supporting Information

© Copyright Wiley-VCH Verlag GmbH & Co. KGaA, 69451 Weinheim, 2011

Abyssomicin Biosynthesis: Formation of an Unusual Polyketide, Antibiotic-Feeding Studies and Genetic Analysis

Elvira M. Gottardi,[a] Joanna M. Krawczyk,[a] Hanna von Suchodoletz,[a] Simone Schadt,[a] Agnes Mühlenweg,[a] Gabriel C. Uguru,[b] Stefan Pelzer,[c] Hans-Peter Fiedler,[d] Mervyn J. Bibb,[e] James E. M. Stach,[b] and Roderich D. Süssmuth*[a]

cbic_201100172_sm_miscellaneous_information.pdf

Supplementary Information

Abyssomicin Biosynthesis: Formation of an Unusual Polyketide Antibiotic - Feeding Studies and Genetic Analysis

Elvira M. Gottardi,[a] Joanna M. Krawczyk[a], Hanna von Suchodoletz[a], Simone Schadt[a], Agnes Mühlenweg[a], Gabriel C. Uguru[b], Stefan Pelzer[c], Hans-Peter Fiedler[d], Mervyn J. Bibb[e], James E. M. Stach[b], Roderich D. Süssmuth[a]

Content:

Content:		1
1	Analytical Data from ^{13}C-labeled substrate feeding experiments	2
1.1	^{13}C-NMR spectra after feeding acetate	2
1.2	^{13}C-NMR spectra after feeding propionate	3
1.3	^{13}C-NMR spectra after feeding glucose	3
2	The abyssomicin biosynthetic gene cluster - Function assignment	5
3	Inactivation studies: Primers and constructs used	7
3.1	Single Crossover Homologous Recombination	7
3.1.1	Primers used for Single Crossover Inactivation	9
3.2	Double Crossover Homologous Recombination by PCR Targeting[3]	9
3.2.1	Procedure	10
3.2.2	Primers used for double crossover inactivation	11
3.3	Double Crossover Homologous Recombination using pK18mob2	12
3.3.1	Procedure	12
3.3.2	Primers	13
3.4	Complementation Experiments	13
3.4.1	Procedure	13
3.4.2	Primers	14
4	Phylogenetic analysis of Aby, Chl, Kij, Tca and Tmn	14
5	References	14

[a] *Prof. Roderich D. Süssmuth, Technische Universität Berlin, Institut für Chemie, Strasse des 17. Juni 124, 10623 Berlin, Germany, suessmuth@chem.tu-berlin.de, Tel. 0049(0)30-314-24205*
[b] *Dr. Jem Stach, School of Biology, Newcastle University, Newcastle-upon-Tyne, NE1 7RU, UK*
[c] *PD Dr. Stefan Pelzer, B.R.A.I.N. Aktiengesellschaft, Zwingenberg, Germany*
[d] *Prof. Dr. Hans-Peter Fiedler, Universität Tübingen, Tübingen, Germany*
[e] *Prof. Dr. Mervyn Bibb, Department of Molecular Microbiology, John Innes Centre, Norwich NR4 7UH, United Kingdom*

1 Analytical Data from ^{13}C-labeled substrate feeding experiments

1.1 ^{13}C-NMR spectra after feeding acetate

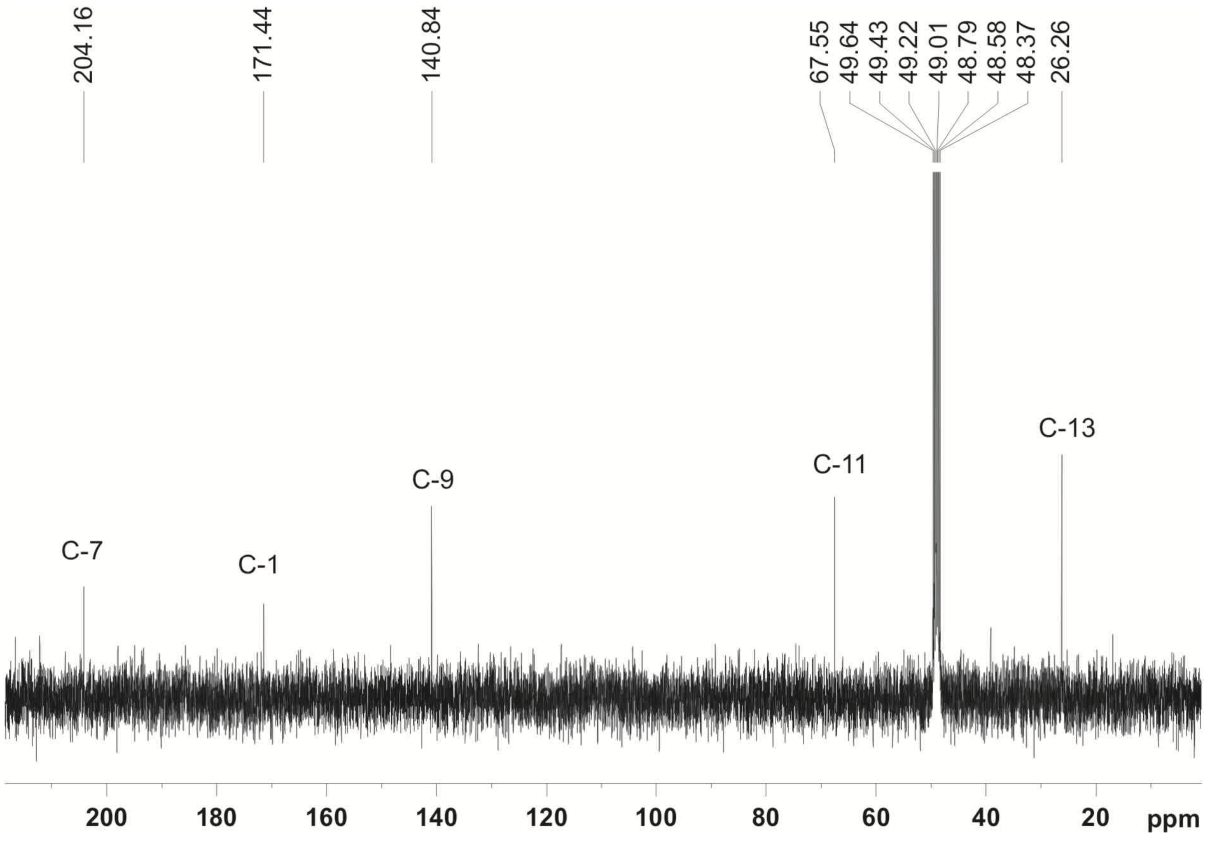

Figure S1: ***^{13}C-NMR-spectrum of atrop-abyssomicin C after feeding of 1-^{13}C-acetate and structural assignment of carbon-atoms.***

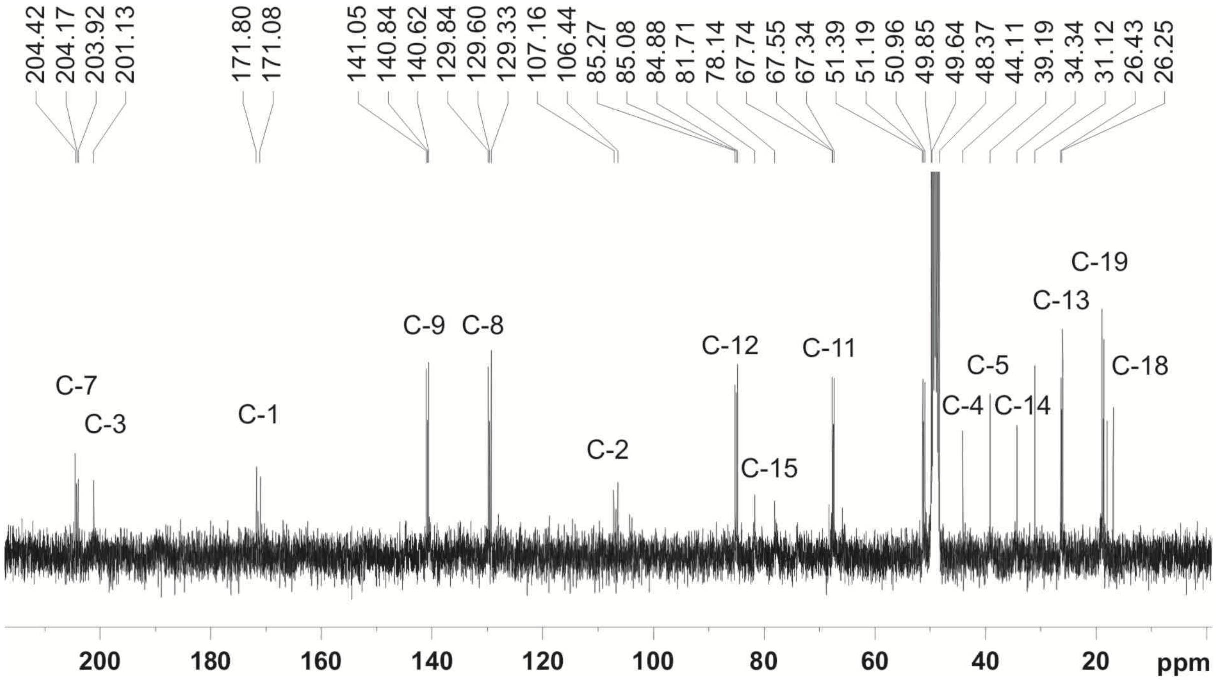

Figure S2: ***^{13}C-NMR-spectrum of atrop-abyssomicin C after feeding of 1,2-^{13}C-acetate and structural assignment of carbon-atoms.***

1.2 ^{13}C-NMR spectra after feeding propionate

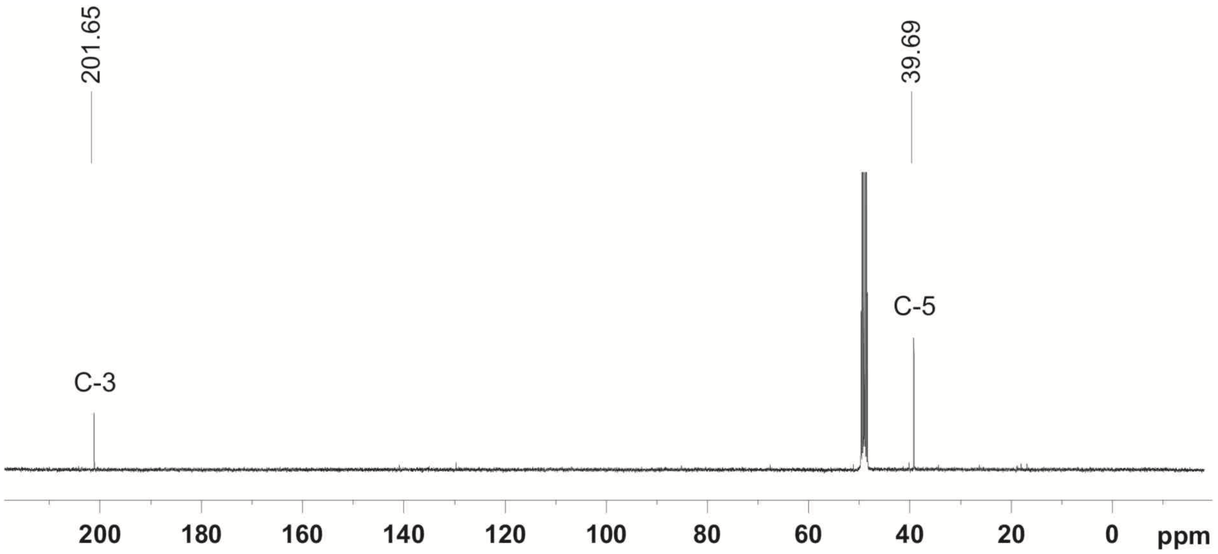

Figure S3: ***^{13}C-NMR-spectrum of atrop-abyssomicin C after feeding of 1-^{13}C-propionate and structural assignment of carbon-atoms.***

1.3 ^{13}C-NMR spectra after feeding glucose

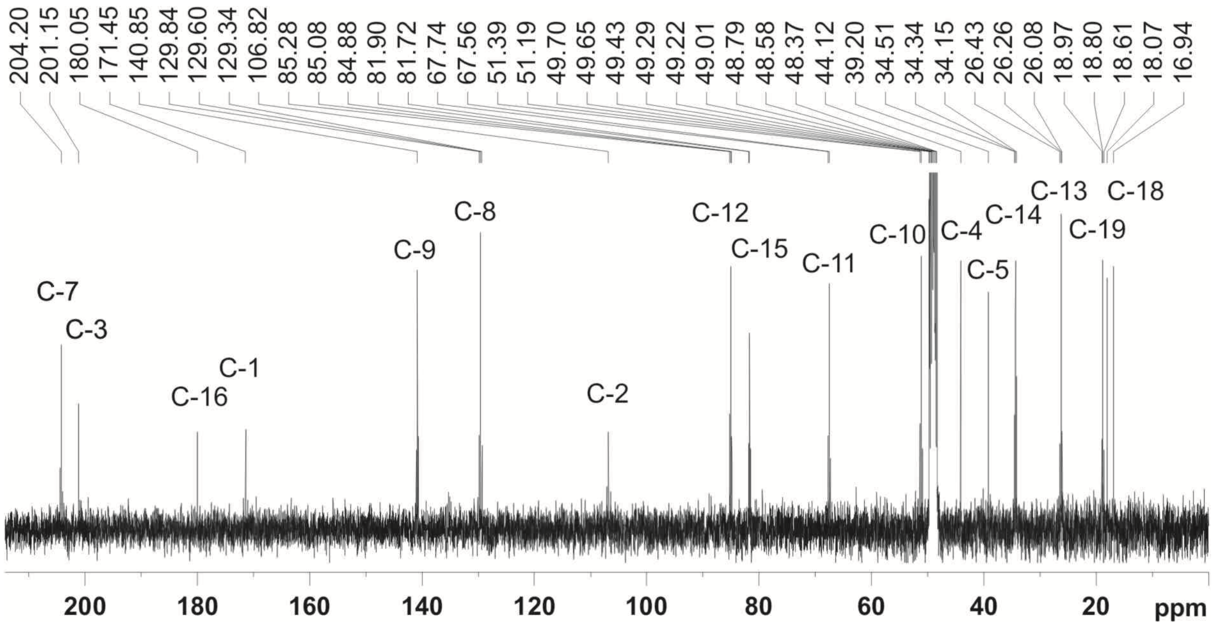

Figure S4: ***^{13}C-NMR-spectrum of atrop-abyssomicin C after feeding of 1,2-^{13}C-glucose and structural assignment of carbon-atoms.***

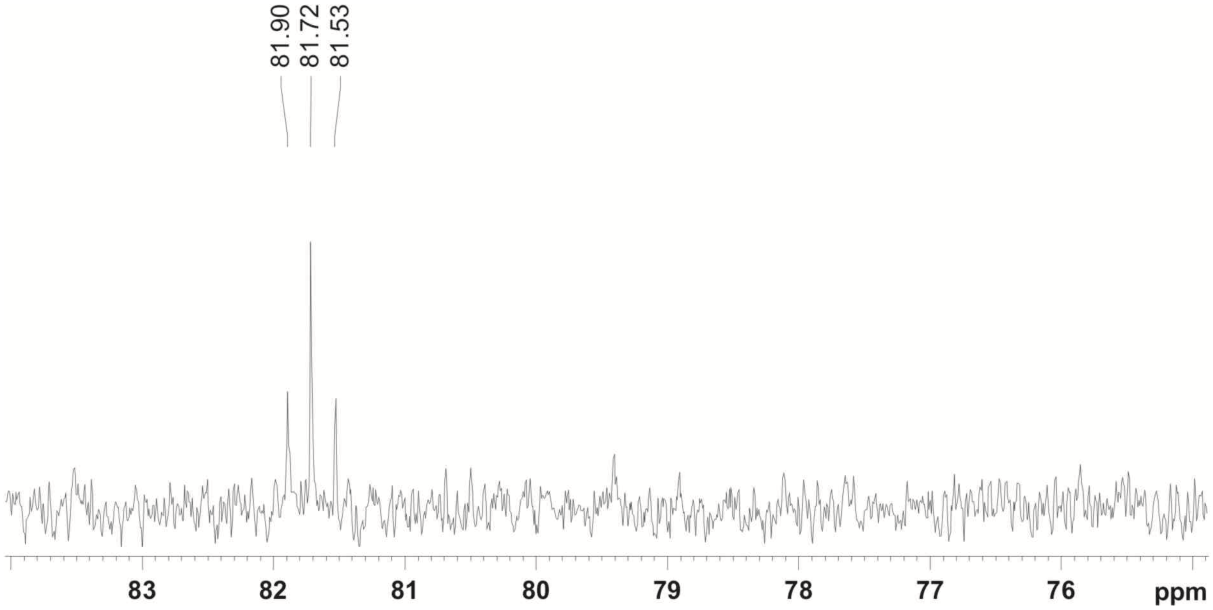

Figure S5: ***^{13}C-NMR-spectrum of atrop-abyssomicin C after feeding of 1,2-^{13}C-glucose: Zoom of signal C-15 of Fig. S4 between 83 ppm and 76 ppm.***

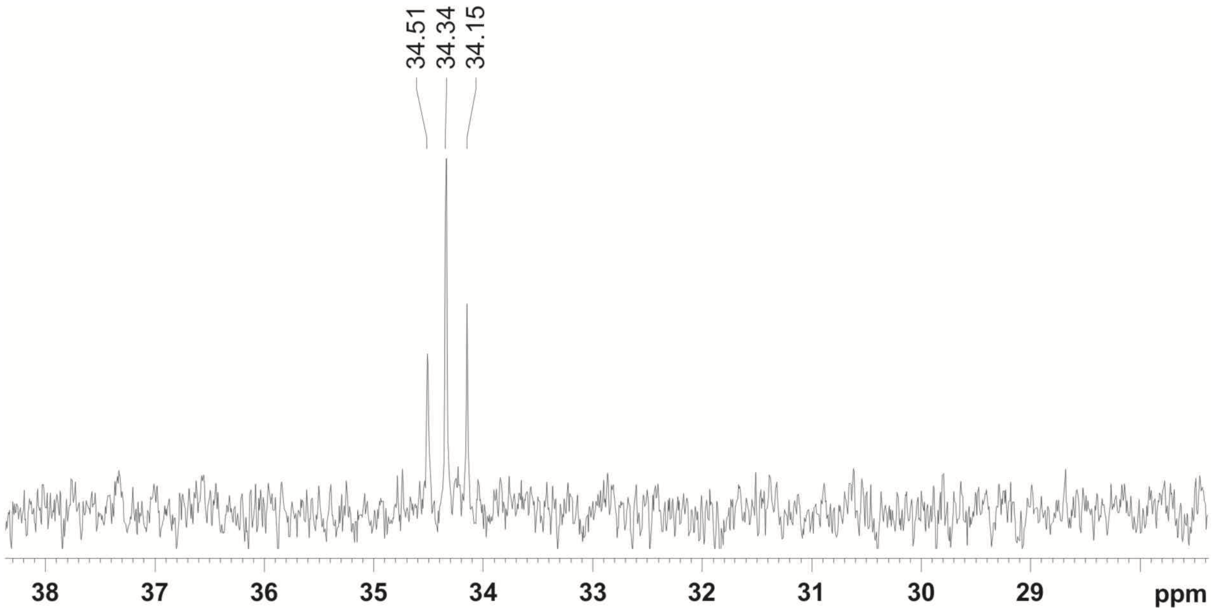

Figure S6: ***^{13}C-NMR-spectrum of atrop-abyssomicin C after feeding of 1,2-^{13}C-glucose: Zoom of signal C-14 of Fig. 4 between 38 ppm and 29 ppm.***

Tab. S1: ^{13}C NMR data of atrop-abyssomicin C ([D$_4$]methanol, 298 K)									
		[1,2-13C2]glucose		[1,2-13C2]acetate		[1-^{13}C]acetate		[1-^{13}C]propionate	
No.	δ(13C) [ppm]	Multiplicity	JC-C [Hz]	Multiplicity	JC-C [Hz]	Multiplicity	JC-C [Hz]	Multiplicity	JC-C [Hz]
1	171.5	d	72.3	d	72.2	s			
2	106.9	d	72.3	d	72.2				
3	201.2	s		s				s	
4	44.7	s		s					
5	39.2	s		s				s	
6	50.0								
7	204.2	d	50.6	d	50.6	s			
8	129.8	d	50.6	d	50.6				
9	140.3	d	43.5	d	43.5	s			
10	51.1	d	43.5	d	43.5				
11	67.6	d	39.5	d	39.5	s			
12	85.1	d	39.5	d	39.5				
13	26.7	d	35.8	d	35.9	s			
14	34.6	d	36.5	s					
15	81.8	d	36.5	s					
16	180.1	s							
17	18.8	d	35.8	d	35.9				
18	16.9	s		s					
19	18.0	s		s					

2 The abyssomicin biosynthetic gene cluster - Function assignment

Table S2: The abyssomicin biosynthetic gene cluster with functions assigned by xblast and InterProScan searches.					
Name	Gene start (bp)	Length (aa)	Proposed Function	Similarity with	Identity/Positives [%]
orfQ	1	208	NUDIX-Hydrolase	*Frankia sp.* EAN1pec	53/66
			putative MutT family protein	*Nocardia farcinica* IFM 10152	43/52
abyR	680	248	transcriptional regulator, SARP family	[*Frankia sp. EUN1f*]	54/71
				Frankia symbiont of *Datisca glomerata*	58/69
abyX	2629	396	cytochrome P450	*Frankia sp.* EAN1pec	66/77
				Streptomyces griseolus	56/70

abyH	5389	889	LuxR family transcriptional regulator	*Frankia alni* ACN14a	33/46
			putative activator	*S. carzinostaticus*	35/45
abyI	5745	252	putative pathway specific activator	*S. longisporoflavus*	50/64
			activator protein	*S. hygroscopicus* ATCC 53653	48/63
abyK	8827	579	YD repeat	*Frankia sp.* EAN1pec	46/57
			RHS repeat-containing protein	*Amycolatopsis mediterranei* U32	36/50
abyA1	8911	341	β-ketoacyl-acyl-carrier-protein synthase I	*Frankia sp.* EAN1pec	68/81
			3-oxoacyl-(acyl-carrier-protein) synthase III	*Streptomyces sp.* NRRL 11266	64/75
abyA2	9933	622	phosphatase and glyceryl transferase	*Streptomyces sp.* NRRL 11266	57/68
			ChlD1	*S. antibioticus*	57/68
abyA3	11798	78	discrete ACP	*Streptomyces sp.* NRRL 11266	58/75
			ChlD2	*S. antibioticus*	53/72
abyA4	12031	251	dehydrogenase catalytic domain-containing protein	*Frankia sp.* EAN1pec;	64/78
			pyruvate/2-oxogluatarate dehydrogenase	*Streptomyces sp.* NRRL 11266	60/73
abyA5	12783	355	hydrolase superfamily dihydrolipoamide acyltransferase-like protein	*Actinomadura kijanata*	54/68
			ChlD4	*S. antibtioticus*	51/65
abyB1	13847	5781	PKS I (module1: KS, ATa, ACP; module 2: KS^Q, ATa, DH, KR, ACP; module 3: KS, ATa, DH, KR, ACP; module 4: KS, ATa, DH, KR, ACP	*S. avermitilis* MA-4680	48/59
abyB2	31269	3645	PKS I (module 5: KS, ATp, DH, KR, ACP; module 6: KS, ATp, DH, ER, KR, ACP)	*Streptomyces sp.* DSM 21069	50/61
abyB3	42203	992	PKS I (module7: KS, ATa, ACP)	*S. antibioticus*	54/65
abyC	45942	230	regulatory protein, TetR	*Frankia sp.* EAN1pec;	60/75
				Brucella abortus biovar 1 str. 9-941	40/56
abyD	46026	475	drug resistance transporter EmrB/QacA subfamily;	*Frankia sp.* EAN1pec;	66/79
			export protein	*S. antibioticus*	44/60
abyE	47524	335	luciferase; alkanal monooxygenase α-chain/	*Frankia sp.* EAN1pec;	60/74
			Flavin utilizing monooxygenases	*Brucella melitensis* 16M	45/64
abyF1	48619	538	ABC transporter oligopeptide binding protein	*Frankia alni* ACN14a;	45/59
			peptide ABC transporter, periplasmic peptide-binding protein	*Klebsiella pneumoniae* 342	40/55
abyF2	50232	311	ABC transporter oligopeptide permease;	*Frankia alni* ACN14a	53/74
			binding-protein-dependent transport systems inner membrane component	*Frankia sp.* CcI3	51/69
abyF3	51164	283	binding-protein-dependent transport systems inner membrane component	*Frankia sp.* EAN1pec	54/70
			oligopeptide ABC transporter permease protein	*Symbiobacterium thermophilum* IAM 14863	45/62

abyF4	52005	539	peptide ABC transporter ATP-binding protein	*Frankia alni* ACN14a	59/71
			ABC transporter ATP-binding protein	*Janthinobacterium sp. Marseille*	52/63
abyV	53621	395	cytochrome P450	*Frankia sp.* EAN1pec	63/72
			cytochrome P450 hydroxylase	*S. avermitilis* MA-4680	51/63
abyW	54771	302	alcohol dehydrogenase zinc-binding domain protein	*S. bingchenggensis* BCW-1	64/76
			oxidoreductase	*Streptomyces hygroscopicus* ATCC 53653	60/74
abyZ	55601	165	NAD(P)H-dependent FMN reductase	*S. clavuligerus* ATCC 27064	73/81
				S. viridochromogenes DSM 40736	75/86
abyT	55726	298	thioesterase	*Nostoc punctiforme* PCC 73102	35/52
			oleoyl-(acyl-carrier-protein) hydrolase	*Haliangium ochraceum* DSM 14365	42/55
orfU	57429	302	alcohol dehydrogenase zinc-binding domain protein	*S. bingchenggensis* BCW-1	64/76
			oxidoreductase	*S. hygroscopicus* ATCC 53653	60/74
orfS	57520	197	transcriptional regulator, TetR family protein	*S. bingchenggensis* BCW-1	59/70
			putative transcriptional regulator	*S. hygroscopicus* ATCC 53653	57/72

3 Inactivation studies: Primers and constructs used

3.1 Single Crossover Homologous Recombination

Internal gene fragments (app. 300bp) were amplified by PCR and cloned into pDrive following the manufacturer's instructions ("PCR cloning kit", Qiagen, Hilden, Germany). After restriction digestion with *Eco*RI, the fragments were ligated with *Eco*RI-digested and dephosphorylated (Antarctic phosphatase, New England Biolabs, Ipswich, MA, USA) pK18mob2[1] and introduced into *E. coli* DH5α by transformation. Clones were confirmed by PCR and/or restriction digestion. Isolated plasmid DNA was introduced into *E. coli* ET12567 containing one of the helper plasmids pUZ8002 or pUB307 by transformation.[2] Intergeneric conjugation followed using protocols described in *Practical Streptomyces Genetics*.[2] After 16 hours of incubation at 28 °C, plates were overlaid with 4ml soft nutrient agar containing 10 µl apramycin (100mg/ml) and 10 µl nalidixic acid (25 mg/ml).[2] After a further 5 days of incubation at 28 °C, colonies were visible which were picked to fresh SGG-agar plates containing 100 µg/ml apramycin and 25 µg/ml nalidixic acid. Insertion into the correct chromosomal location was confirmed using a 3-primer system (the orientation of the insert in the vector was arbitrary, reflecting the cloning procedure): Two primers (confirmation primers 1 and 2) corresponded to the genome sequence of *Verrucosispora* AB-18-032 flanking the amplified region, while one primer corresponded to vector sequences (confirmation primer vector; see Figure S7). The wild type genome would yield a PCR fragment from confirmation primers 1 and 2 that would be shorter than the one expected for a mutant clone (confirmation primer 1 or 2 with confirmation primer vector; Figure S7 and Figure S8). Final confirmation was achieved by sequencing of the PCR-product. A second gene deletion procedure (SOEing)[3] was used to generate in-frame deletions (ca. 90% of the coding region of individual genes) for *abyR*, *abyI* and *abyT*. Two sets of primers were used to generate overlapping fragments that were spliced together by PCR to form the gene deletion cassette. The deletion cassettes were cloned into the non-integrating vector pKC1132[4] that had been digested with EcoRI and HindIII. Resulting constructs were used to transform *Verrucosispora* AB-18-032 by conjugation as described above. Double

crossover mutants (see below) were selected by screening for loss of apramycin resistance followed by PCR and sequencing confirmation. The primers used for SOEing are given in Table 2.

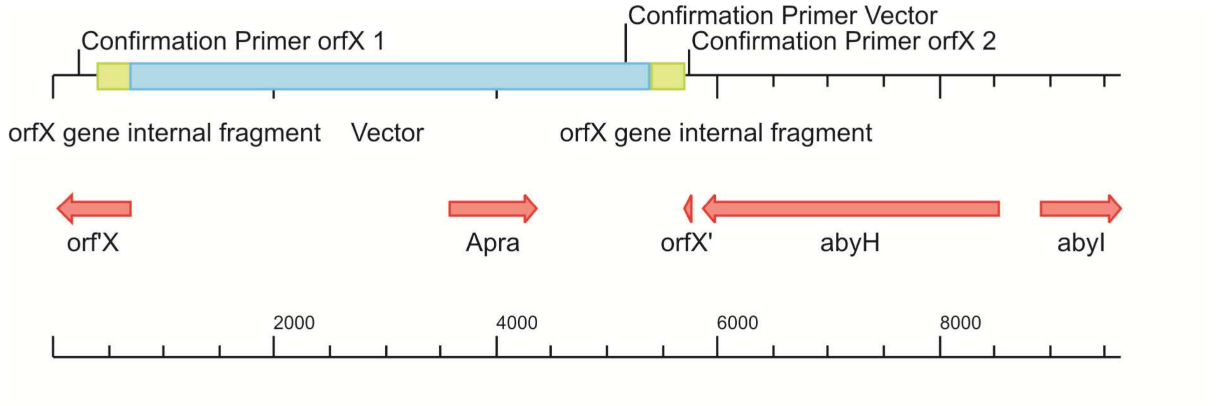

Figure S7: Scheme of genomic DNA sequence with inserted pK18mob2::abyXfragment (length 9645 bps).

Table S3: Primers for generation and confirmation of single crossover ("SXO") mutants	
Primers for insertion-fragment and confirmation primers	
SXO abyX fw	TCGAGACGGGCGCTGACG
SXO abyX rev	CCGCCCTGCGCGAGAATG
SXO orfV fw	AGAGCCACTCCGAGCGCCAACG
SXO orfV rev	CTCCTCGACGAGGTGGTTC
SXO abyC fw	CGTCGAACAGGTCGGGAAAG
SXO abyC rev	TGGCGAAGCCGACCATCTAC
confirmation primer vector	CGCGCCATTCGCCATTCAGG
abyX confirmation primer 1	CCTGTGCGGTGCTCGAAC
abyX confirmation primer 2	ATGACCCGCACCTGTCCC
orfV confirmation primer 1	CGCGGTCCGCAACTTCATCG
orfV confirmation primer 2	TGCCGCAGGATCTCCTCCAC
abyC confirmation primer 1	GAGCGCCTCGGTGGCCTGGTTC
abyC confirmation primer 2	GTGTTCGCCCCGCGAGGGCTACG
Primers for SOEing (restriction site underlined)	
abyI_1F	GCCTGCGAATTCGGACCTGTGGGTCCTTGATG
abyI_1R	GAGGAATCTAGATAATTCGTAGCGCATTGCTG
abyI_2F	GAATTATCTAGAGAGGAACTCGGGTTGGAG
abyI_2R	GAATTCAAGCTTAACCCGTTCCGCTACCTG
abyR_1F	GAGCGGGGATCCGGGAAACCTCGTATCGAAGC
abyR_1R	GTTGGCTCTAGAATGAGTTCGTCGGTGGTGAC
abyR_2F	ACTCATTCTAGAGTTGGCGGCGTACCAGTC

abyR_2R	GGACAAGCTTCCAAGGACATCTCCAACAGC
abyT_1F	CTGACCTGGATCCGGTCGACCTGGCACTGTTG
abyT_1R	CGGTCATCTAGAGTCCAGGTAACCGGTCGATC
abyT_2F	CTGGACTCTAGACGGTCACCTGTTCCACCT
abyT_2R	CCGTCCGAAGCTTGTAGGTGGGGTCGTCGTG

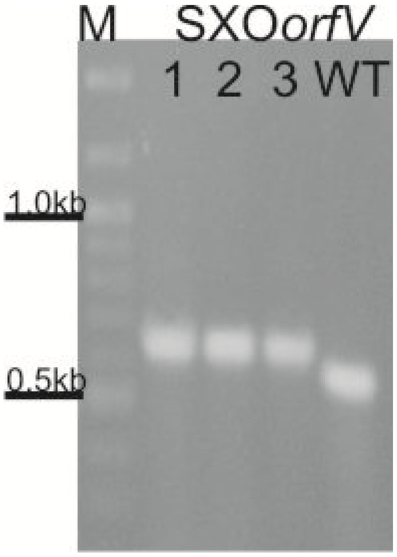

Figure S8: Example of confirmation of insertional inactivation: Agarose gel of PCR products generated with confirmation primers (SXOorfV = single crossover mutants of orfV; 1-3 = mutant samples; WT = wild type Verrucosispora sp. AB-18-032; M = bp length marker).

3.1.1 Primers used for Single Crossover Inactivation

Table S4: Lengths of PCR fragments wild type vs. single crossover mutant		
Gene	Verrucosispora::pK18_genefragment	WT
abyX	689 or 568bp	504bp
abyC	>462bp	357bp
orfV	732 or 682bp	584bp

3.2 Double Crossover Homologous Recombination by PCR Targeting[3]

To achieve in-frame deletion of genes, cosmid-clones were mutagenised according to protocols described by Gust et al.[5] pOJ436[2] contains an *oriT* as well as a ΦC31 attachment site, so a modified protocol was used. Primers for amplification of the streptomycin resistance cassette were designed so that only the antibiotic resistance gene plus promoter were amplified and not the whole cassette containing FRT and *oriT*. Restriction sites were introduced between the streptomycin resistance cassette primer and the gene-specific

homologous sequence of 39nt. In the case of cosmid 49 (A-3 – A4), *Nde*I was used, for cosmid 17 (C1) and for cosmid 45 (C2 and C3), a combination of *Nhe*I and *Xma*JI were used.

3.2.1 Procedure

pIJ778 was digested with *Eco*RI and *Hin*dIII and gel-purified twice (QiaexII gel extraction kit, Qiagen). PCR was carried out following the protocol of Gust et al. (for primers see Table S). The PCR product was purified using the Illustra GFX PCR DNA and Gel Band Purification Kit (GE Healthcare). Cosmids 17, 45 and 49 were introduced into *E. coli* BW25113/pIJ790 by electroporation. After PCR-targeting of the cosmids, clones were analyzed by colony-PCR (using primers lying approximately 100bp to each side of the homologous regions, see Table S). Confirmed positive clones were grown in LB medium at 30°C and 200rpm. After isolation the mutagenised, streptomycin resistant cosmid was digested with *Nde*I or *Nhe*I/*Xma*JI, respectively. Religation followed and the ligation mix was desalted and introduced into *E. coli* DH5α by transformation. Streptomycin sensitive clones were analyzed by colony-PCR (primers Table S). The confirmed streptomycin sensitive cosmid was introduced into *E. coli* BW25113/pIJ790 and mutagenised with a PCR product obtained with primers for the streptomycin resistance cassette containing two 39nt extensions homologous to the flanking regions of the ΦC attachment site *attB* and the integrase. Confirmed clones (designated $\Delta\Phi$C31) lacked the ability to integrate into the genome of Verrucosispora via *attB*/*attP* site-specific integration. After introduction into *E. coli* ET12567/pUZ8002, intergeneric conjugation was carried out. After 5-7 days, exconjugants were picked to SGG plates containing 100µg/ml apramycin and 25µg/ml nalidixic acid (SGG: 1% dextrin, 1% glucose, 1% glycerol, 0.25% corn steep powder (Marcor, Carlstadt, NJ, USA), 0.5% peptone, 0.2% yeast extract, 0.1% NaCl, 0.3%, CaCO3 0.3% in tap water (1 L), adjusted to pH 7.3 prior to sterilization) plates and grown for 3-5 days after which several clones were chosen and transferred to fresh SGG-agar plates for three passages. Subsequently, spores were harvested from 7-10 day old patches and a spore dilution distributed on fresh SGG agar plates. After 5-7 days, individual colonies were visible and were picked onto SGG-Apra agar and SGG-agar. 2-3 day old apramycin sensitive (Apra^S)clones were analyzed by colony PCR (a small amount of mycelium was dispersed in 100µl water and incubated for 10min at 100°C; after cooling on ice and centrifugation 3-10µl of the supernatant were used as template in the PCR; for typical results see Figure S9). PCR-products were verified by sequencing. Several exconjugants per gene knockout were analyzed, and three individual clones of each mutant were identified and tested for production.

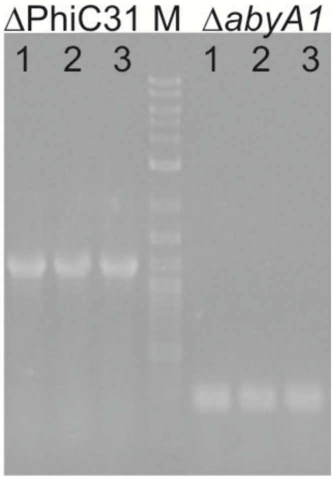

Figure S9: Colony-PCR products with confirmation primers. $\Delta\Phi$C31 has a length of app. 1.2kb, ΔabyA1 a length of app. 300bp.

3.2.2 Primers used for double crossover inactivation

Table S5: ReDirect Primers for PCR-directed mutagenesis	
abyI Red up	GCAATGCGCTACGAATTACTCGGACCACTACGACTGGTGCATATGCTTATTTGCCGACTACCTTG
abyI Red down	GCCTGCCCTTCAGCAGGCTTGGGCTACCTCCCGCCGCCGCATATGTATCACCACCGACTATTTGC
abyA1 Red up	CCGCCTCACGCCGACCCCCTCGGGAGGACTGCAATGACCCATATGTATCACCACCGACTATTTGC
abyA1 Red down	ACCCGGTGGCGGACGGGGCCGCGGCACCCGCCGTCACAGCATATGCTTATTTGCCGACTACCTTG
abyA2 Red up	GATCAAGTCGGGCTTCGCGTTGGAGGGCCTGTGACGGCGCATATGTATCACCACCGACTATTTGC
abyA2 Red down	GGGTGACGAAGTCGTGGACGGATCGCAGGGTGCTCACGGCATATGCTTATTTGCCGACTACCTTG
abyA3 Red up	GTTGACCGGCTCCTGGCCCCCCGGAGGAACCCCGTGAGCCATATGTATCACCACCGACTATTTGC
abyA3 Red down	GCCGGCGCTCGCGGGCGACGGGGGAGACGCTGGTCACCGCATATGCTTATTTGCCGACTACCTTG
abyA4 Red up	CCTGGGCGGGATCTACGCGACGGCGGTGGATCGGTGACCCATATGTATCACCACCGACTATTTGC
abyA4 Red down	GGACGTACTGCTTCAGCTCTGCGACGTCGTTGCTCATGCCATATGCTTATTTGCCGACTACCTTG
abyC Red up	ACCAGCCCCGGCCGGGTCGGCGCGGCCCGCCGGGGCTCACCTAGGTATCACCACCGACTATTTGC
abyC Red down	AGTTCAGTTCGGCGCACTGCACGGTAACATGGCCGAATGGCTAGCCTTATTTGCCGACTACCTTG
abyD Red up	TCTATCGAACCGGCCCGACAAAAACCGGAGACGGCCATGCCTAGGTATCACCACCGACTATTTGC
abyD Red down	CTCGGGGGCGCCGACCGGCCCCGGGTTTGGGGGTCGTCAGCTAGCCTTATTTGCCGACTACCTTG
abyE Red up	AGTCGACCGGCCCCGTACCCACCCAGAGAGGCCCCGATGCCTAGGTATCACCACCGACTATTTGC
abyE Red down	GCGCCTAAAGCTGGCCTAAACCCGCAGGTCGCGCGCCTAGCTAGCCTTATTTGCCGACTACCTTG
PhiC31Integr up	CTGGGTGGGTTACACGACGCCCCTCTATGGCCCGTACTGTATCACCACCGACTATTTGC
PhiC31Integr down	CCGTCTCAGCGCCTAACAGGCTTCCCGGGTGTCTCGCTACTTATTTGCCGACTACCTTG

Table S6: Confirmation Primers	
abyI Best. 1	CGGCGAAATACCATCCTGAC
abyI Best. 2	ACGTGCGGACGAGTAAGTG
abyA1 Best. upstream	CCGTCCGCTCGTTCATGG
abyA1 Best. downstream	CTGCGGTACAGCTCGGACAG
abyA2 Best. upstream	GACAGCGGGCGCTGGGTGTG
abyA2 Best. downstream	CGGCCGGGAGCGAGATGGAC
abyA3 Best. upstream	CGCCGGAACACGTCCAGTTG
abyA3 Best. downstream	CGCACGGCCTCCAGGAAGAAG
abyA4 Best. upstream	CGCCACCGGGCGGTCCATCTC
abyA4 Best. downstream	GCCGGCGCGGATCCACTGTGTC
abyC Best. upstream	TGTCGCCGCTGGCGCTGTTC
abyC Best. downstream	GTCGGGCCGGTTCGATAG
abyD Best. upstream	GGTGTCGCGCTCAGTCATTC
abyD Best. downstream	GACGTCGAGGACGGATAC
abyE Best. upstream	ACGCCCGCAGCCCGTCACCC
abyE Best. downstream	CGCGGTGGCGGCGACCAGGAG
PhiC31 Best. upstream	CGCCCACTGCCTTCAGAC
PhiC31 Best. downstream	CCGCTGTGCTGTGGTGAC

3.3 Double Crossover Homologous Recombination using pK18mob2

Deletion mutants for genes *abyE* and *abyK* were made by double crossover recombination using pK18mob2.

3.3.1 Procedure

Generation of an *abyE* gene replacement mutant

Two 1.4 kb fragments of the flanking regions of *abyE* were amplified by PCR using the primer pairs *abyE*_EcoRI_for, *abyE*_EcoRV_rev, *abyE*_EcoRV_for and *abyE*_XbaI_rev. The resulting PCR fragments *abyE*_EcoRI_EcoRV and *abyE*_EcoRV_XbaI were cloned together into pUC21 to generate pUC21_Δ*abyE*. The insert of pUC21_Δ*abyE* was excised with *Eco*RI/*Xba*I and cloned into pK18mob2[1]. Transfer to *Verrucosispora* AB-18-032 was performed as described in 2.1. Apramycin-resistant colonies were obtained and single cross-over events were confirmed by isolation of genomic DNA and PCR analysis with the primer pair *abyE*_proof_for / *abyE*_proof_rev. Integrants were grown without apramycin selection to allow for second cross-over recombination. Apramycin-sensitive clones were confirmed by PCR for the deletion of the internal fragment of *abyE*.

Generation of an *abyK* gene replacement mutant

Similar to the generation of the *abyE* inactivation construct, flanking regions of *abyK* were amplified with the primer pairs *abyK*_EcoRI_for / *abyK*_EcoRV_rev and *abyK*_EcoRV_for / *abyK*_XbaI_rev and cloned into pK18mob2. Transfer to *Verrucosispora* AB-18-032, generation and verification of the double crossover mutant was performed as described above using the primers *abyK*_proof_for / *abyK*proof_rev.

Generation of an *abyX* gene replacement mutant

Similar to the generation of the *abyE* inactivation construct, flanking regions of *abyX* were amplified with the primer pairs *abyX*_EcoRI_for / *abyX*_EcoRV_rev and *abyX*_EcoRV_for / *abyX*_XbaI_rev and cloned into pK18mob2. Transfer to *Verrucosispora* AB-18-032, generation and verification of the double crossover mutant was performed as described above using the primers *abyX*proof_for / *abyX*proof_rev.

3.3.2 Primers

Table S7: Primers for double crossover homologous recombination using pK18mob2	
abyE_EcoRI_for	GACCGCTGAATTCGACGCCACACTG
abyE_EcoRV_rev	CCGTGATATCGCTCCAGACCGGAAC
abyE_EcoRV_for	GGCGCTGGATATCCTGCCCGAGCTG
abyE_XbaI_rev	CTGCCCGTCTAGATTCCGTTGGTCGCGTTCC
abyE_proof_for	CTCCTTCGACCAAGCCTTCTG
abyE_proof_rev	GGAGCAGCAGAATGGCTGTC
abyK_EcoRI_for	GGGCAGGAATTCGCTACGAATTAC
abyK_EcoRV_rev	CACTTCCTGATATCCCGCATCTACAAC
abyK_EcoRV_for	CGAGCGATATCTCCGGGTGGTAG
abyK_XbaI_rev	CTGCGGTCTAGATCGGACAGCTCAG
abyK_proof_for	TGATGAACGGAATGCCGGTGCCGAGAAAC
abyKproof_rev	GCGGCGAGTTGCCCGAGACCAC
abyX_EcoRI_for	GGGCAACGAATTCACCAGCCGGTATCGC
abyX_EcoRV_rev	AACCGATATCCGGCCGCCTTC
abyX_EcoRV_for	GGACAGATATCGCCGCGATCAGC
abyX_XbaI_rev	CGGCGCTCTAGACGAGTGCCTGCTGG
abyXproof_for	GGTACACGCCCTGCACCGAG
abyXproof_rev	GTTCCCGATGACCCGCACCTG

3.4 Complementation Experiments

3.4.1 Procedure

To complement the deletion mutants Δ*abyI* and Δ*abyK*, the genes were amplified by PCR using primers *abyI*_fw and *abyI*_rev (*abyK*_HindIIIfor / *abyK*_SpeI_rev). The PCR-products were purified using Invitek-columns and digested with *Eco*RI and *Xba*I. The product was ligated with *Eco*RI and *Xba*I-digested rs pSETermEΔHindIII and pUWLoriT, respectively. The constructs were introduced by electroporation into *E.coli* ET12567 + pUZ8002, followed by standard conjugation using spores from deletion mutants Δ*abyI* and Δ*abyK* using methods described in 2.1.

3.4.2 Primers

Table S8: Primers for Complementation Experiments	
abyI_fw	AAACCGAATTCTCGGAGGCAGCAATG
abyI_rev	CCCGCTCTAGATCAGCAGGCTTGGGCTAC
abyK_HindIII_for	CCAAAAGCTTGGGAGGCGACTGTGGCGCTCCGTC
abyK_SpeI_rev	GCGGTCACTAGTCCAGTCGCTCAGTCATTGTCG

4 Phylogenetic analysis of Aby, Chl, Kij, Tca and Tmn

A

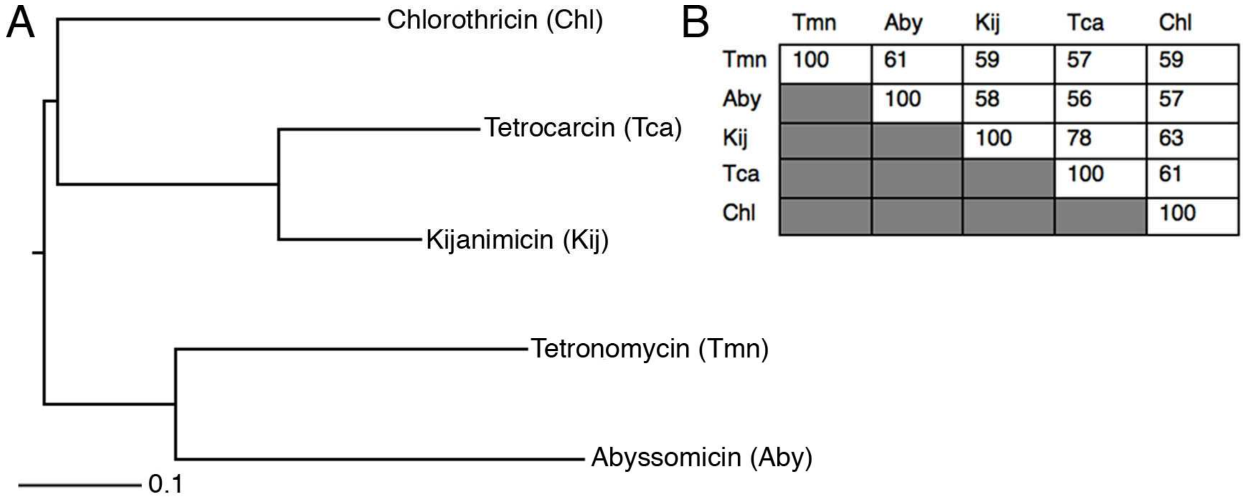

B

	Tmn	Aby	Kij	Tca	Chl
Tmn	100	61	59	57	59
Aby		100	58	56	57
Kij			100	78	63
Tca				100	61
Chl					100

Fig. S10: ***A) Phylogenetic analysis of Aby, Chl, Kij, Tca and Tmn (see main paper for references) proteins involved in tetronic acid ring formation. Five proteins homologous to ChlM and ChlD1-D4 from each biosynthetic cluster were concatenated in the above order and aligned. Phylogenetically uninformative sites were removed from the alignment using Gblocks.[6] The inferred maximum-likelihood phylogenetic tree was produced using PHYLIP.[7] The scale bar indicates the branch length corresponding to 0.1 amino acid substitutions per site. B) Percentage identity table generated from the concatenated alignment.***

5 References

1. Pelzer, S., Dorsch, H. & Spellig, T. A gene cluster encoding an efomycin polyketide synthase from a Streptomycete. in Ger. Offen. 40 (Germany, 2006).
2. Kieser, T., Bibb, M.J., Buttner, M.J., Chater, K.F., Hopwood, D.A. Practical Streptomyces Genetics, 613 (The John Innes Foundation, Norwich, 2000).
3. Horton, R.M., Hunt, H.D., Ho, S.N., Pullen, J.K. & Pease. L.R.. Engineering hybrid genes without the use of restriction enzymes: Gene splicing by overlap extension. Gene. 77, 61-8 (1989)
4. Bieman M, Logan, R., O'Brien, K., Seno, E.T., Rao, R.N. & Schoner, B.E. Plasmid cloning vectors for the conjugal transfer of DNA from *Escherichia coli* to *Streptomyces* spp. *Gene.* 116, 43 – 9. (1992).
5. Gust, B., Challis, G.L., Fowler, K., Kieser, T. & Chater, K.F. PCR-targeted *Streptomyces* gene replacement identifies a protein domain needed for biosynthesis of the sesquiterpene soil odor geosmin. Proc. Natl. Acad. Sci. U. S. A. 100, 1541-6 (2003)
6. Talavera, G. & Castresana, J. Improvement of phylogenies after removing divergent and ambiguously aligned blocks from protein sequence alignments. *Systematic Biol* 56, 564-77. (2007).
7. Felsenstein, J. PHYLIP - Phylogeny Inference Package (Version 3.2). Cladistics. 5, 164-6 (2005).
